# The continuum of prevention and heart failure in cardiovascular medicine: A joint scientific statement from the Heart Failure Society of America and the American Society for Preventive Cardiology

**DOI:** 10.1016/j.ajpc.2025.101069

**Published:** 2025-08-13

**Authors:** Anuradha Lala, Craig Beavers, Vanessa Blumer, LaPrincess Brewer, Diana De Oliveira-Gomes, Sandra Dunbar, Hannah Every, Richard Ferraro, Bonnie Ky, James Januzzi, Francoise Marvel, Robert Mentz, Erin Michos, Jagat Narula, Khuram Nasir, Pradeep Natarajan, Lori Ann Peterson, Fatima Rodriguez, Michael D. Shapiro, Jenna Skowronski, Randall C. Starling, Pam Taub, Ryan J. Tedford, Quentin Youmans, Shelley Zieroth, Martha Gulati

**Affiliations:** a The Mount Sinai Fuster Heart Hospital and Department of Population Health Science, Icahn School of Medicine, United States; b University of Kentucky College of Pharmacy, Lexington, KY, United States; c Inova Schar Heart and Vascular Institute, Inova Fairfax Medical Campus, Falls Church, VA, United States; d Department of Cardiovascular Medicine, Mayo Clinic, Rochester, MN, United States; e Department of Internal Medicine, University of Texas Southwestern Medical Center, Dallas, United States; f Nell Hodgson Woodruff School of Nursing, Emory University, Atlanta, GA, United States; g Johns Hopkins Hospital, Department of Medicine, Division of Cardiology, Baltimore, MD, United States; h Department of Medicine, Division of Cardiology, Perelman School of Medicine at the University of Pennsylvania, Philadelphia, PA, United States; i Abramson Cancer Center, Perelman School of Medicine at the University of Pennsylvania, Philadelphia, PA, United States; j Department of Biostatistics, Epidemiology & Informatics, Perelman School of Medicine at the University of Pennsylvania, Philadelphia, PA, United States; k Division of Cardiology, Massachusetts General Hospital, Baim Institute for Clinical Research, Harvard Medical School, Boston, MA, United States; l Ciccarone Center for the Prevention of Cardiovascular Disease, Division of Cardiology, Department of Medicine Johns Hopkins University School of Medicine Baltimore Maryland, United States; m Duke Clinical Research Institute, Durham, NC, United States; n Division of Cardiology, Department of Medicine, The University of Texas Health Science Center at Houston, McGovern Medical School, Memorial Hermann Hospital, Houston, TX, United States; o Houston Methodist DeBakey Heart & Vascular Center, Houston Methodist, Houston, TX, United States; p Department of Medicine, Harvard Medical School and Center for Genomic Medicine and Cardiovascular Research Center, Massachusetts General Hospital, Boston, MA, United States; q Program in Medical and Population Genetics, Broad Institute of Harvard and MIT, Cambridge, MA, United States; r Department of Cardiology, Mayo Clinic, Phoenix, AZ, United States; s Division of Cardiovascular Medicine, Department of Medicine, and the Stanford Cardiovascular Institute, Stanford University, Palo Alto, CA, United States; t Center for Prevention of Cardiovascular Disease, Section on Cardiovascular Medicine, Wake Forest University School of Medicine, Winston-Salem, NC, United States; u Division of Cardiology, University of Pittsburgh, Pittsburgh, PA, United States; v Department of Cardiovascular Medicine, Cleveland Clinic, Kaufman Center for Heart Failure Treatment and Recovery, Cleveland, OH, United States; w Division of Cardiovascular Medicine, UC San Diego School of Medicine, La Jolla, CA, United States; x Department of Medicine, Division of Cardiology, Medical University of South Carolina, Charleston, United States; y Division of Cardiology, Department of Medicine, Northwestern University Feinberg School of Medicine, Chicago, IL, United States; z Section of Cardiology, Max Rady College of Medicine, University of Manitoba Winnipeg, Manitoba, Canada; aa Barbra Streisand Women’s Heart Center, Smidt Heart Institute at Cedars Sinai Medical Center, Los Angeles, California and The Baim Institute for Clinical Research, Boston, CA, United States

**Keywords:** Heart Failure Prevention, Continuum of Prevention, Risk Stratification, Lifestyle Interventions, Social Determinants of Health, Advanced Therapies (Tertiary Prevention)

## Abstract

Heart disease is the leading cause of death worldwide, with heart failure (HF) recognized as its most severe and debilitating manifestation. Though remarkable advancements have led to the establishment of life-saving and quality-of-life-enhancing medical and device-based therapies for HF, HF-related mortality trends have increased over the past decade. To combat this worldwide epidemic, care must evolve so that preventative recommendations are not siloed from HF management. Prevention must be prioritized more broadly, not only in the early detection and deterrence of HF, but across a patient’s lifespan in conjunction with therapeutic intervention. Members of the Heart Failure Society of America and the American Society for Preventive Cardiology created this joint Societal Scientific Statement on the Prevention of Heart Failure to emphasize the links between cardiovascular disease prevention and HF and offer a conceptual roadmap along which to consider all aspects of preventative care. This includes primary prevention to reduce the burden of HF, secondary prevention to reduce the impact of HF among those with an established diagnosis of HF, and tertiary prevention, which encompasses the management of risk factors in patients who require advanced therapies, including durable mechanical circulatory support and heart transplantation.

**Summary**

Prevention is an important aspect of heart failure that is not currently being prioritized. Members of the Heart Failure Society of America and the American Society for Preventive Cardiology created this joint Societal Scientific Statement on the Prevention of Heart Failure to emphasize the links between cardiovascular disease prevention and HF.

Alt-text: Unlabelled box

## 1 Introduction

Despite major scientific advancements, heart disease remains the leading cause of death worldwide. In the United States (U.S.), an estimated 26 million adults are affected by cardiovascular disease (CVD), with heart failure (HF) recognized as its most severe and debilitating manifestation [1]. HF is the most commonn cause of hospitalization among older adults in the United States, and is associated with marked decrements in quality of life, high rates of mortality, and annual associated costs that well surpass 30 billion dollars [2,3]. One in four U.S. adults will develop HF during their lifetime, and an additional 33% of the general population are considered at-risk [2]. Though remarkable advancements have led to the establishment of life-saving and quality-of-life-enhancing medical and device-based therapies for HF, HF-related mortality trends have increased since 2011 [4]. These staggering and sobering statistics compel a paradigm shift from siloed models of preventive care considered separately from HF management to one that prioritizes prevention more broadly, not only in the early detection and deterrence of HF, but across a patient’s lifespan alongside therapeutic intervention.

While on the surface preventive cardiology and HF may seem like disparate entities, the two specialties exist across a shared spectrum. In fact, the most recent iteration of the American Heart Association (AHA)/American College of Cardiology (ACC)/Heart Failure Society of America (HFSA) HF guidelines place special emphasis on HF prevention [3]. Changes were made in the nomenclature to underscore the importance of identifying and treating persons “at risk” for HF or with “pre-HF”, as well as the essential role of lifestyle interventions throughout the patient journey [3]. Additional opportunities for timely risk stratification and intervention include assessment of genetic risk, cardiac rehabilitation (CR), and holistic considerations to nurture patient well-being, as well as integration of potential digital health and cardiac devices to enhance patient monitoring and empowerment.

The purpose of this joint Scientific Statement from the HFSA and the American Society for Preventive Cardiology (ASPC) is two-fold: first, to emphasize the inextricable links between CVD prevention and HF regardless of ejection fraction (EF), and second, to offer a conceptual framework (Central Figure) with which to consider prevention as critical not only in the primary capacity to reduce the burden of incident HF (primary prevention), but also in an ongoing fashion for patients with established diagnoses of HF across the EF spectrum (secondary prevention), as well as for those patients who require advanced therapies including durable mechanical circulatory support (MCS) and heart transplantation (tertiary prevention). Overlapping recommendations in prevention and HF cardiology will be highlighted, while outlining gaps in knowledge and providing the panel’s recommendations to better link CVD prevention and HF care. This shared document emphasizes the need for close collaboration between HF specialists and preventive cardiologists, in addition to other subspecialists within and outside of cardiology, to facilitate and promote the implementation of multimodal CVD prevention to combat incident as well as existing HF.

**Figure:**
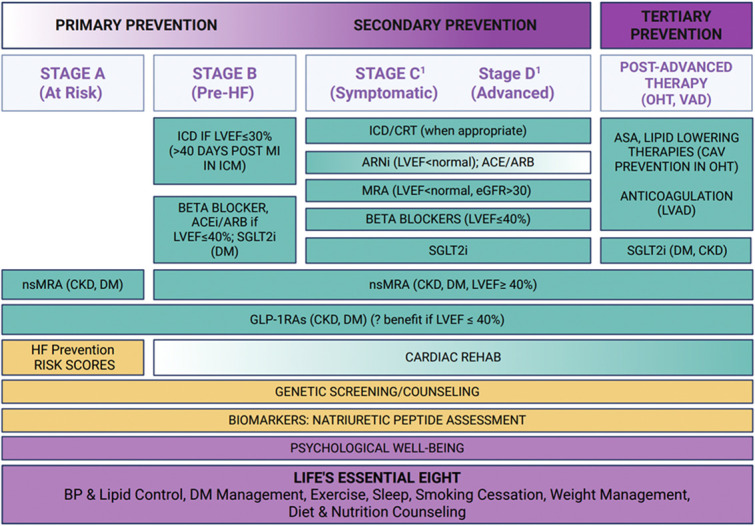
Central Figure: Depictions for treatment included herein are not exhaustive. Blood pressure and lipid management for example are included under Life's Essential 8 as outlined, however detailed discussion of their management are not included here however. Please refer Table 1. The prevention of heart failure (HF) should occur at all stages, including identification of persons at risk of HF to those living with HF, to those who have gone on to receive heart replacement therapies. Many aspects of HF prevention are common irrespective of HF Stage. ***This central figure is not exhaustive (i.e., does not include discussion of traditional hypertension and lipid management), but these are applicable per current guidelines (see below) and are further outlined in Table 1. Heidenreich, P. A. et al. 2022 ACC/AHA/HFSA Guideline for the Management of Heart Failure: Executive Summary. Journal of Cardiac Failure, Volume 28, Issue 5, 810 – 830. ACEi= angiotensin-converting enzyme inhibitors; ARB=angiotensin II receptor blocker; ARNi=angiotensin receptor neprilysin inhibitor; BP=blood pressure; CAV=coronary allograft vasculopathy; CKD= chronic kidney disease; DM= diabetes GLP-1RA= glucagon-like peptide-1 receptor agonists; HF= heart failure; ICD=implantable cardioverter-defibrillator; ICM= ischemic cardiomyopathy; LVEF= left ventricular ejection fraction; MRA= mineralocorticoid receptor antagonists; NICM= non-ischemic cardiomyopathy OHT=orthotopic heart transplant; SGLT2i= sodium-glucose cotransporter-2 inhibitor; VAD= ventricular assist device.

## 2 Definition and staging of heart failure

HF is a complex and progressive condition categorized via a staging system A-D that denotes disease progression [5]. Patients range from being “at risk” for developing HF to having refractory symptoms, facing increasing morbidity and mortality as the disease advances. Thus, preventing the progression from being at risk to pre-HF to symptomatic/clinical HF is crucial for improving patient outcomes [6].

Over the past two decades, the demographic profile of patients living with HF has evolved, influenced by advancements in biomarker-based disease detection, improved management of ischemic heart disease, and the expansion of guideline-directed medical therapy (GDMT). However, this progress has also been accompanied by increasingly complex hemodynamic profiles and a higher prevalence of comorbid conditions, further complicating disease management.

**Fig. 1:**
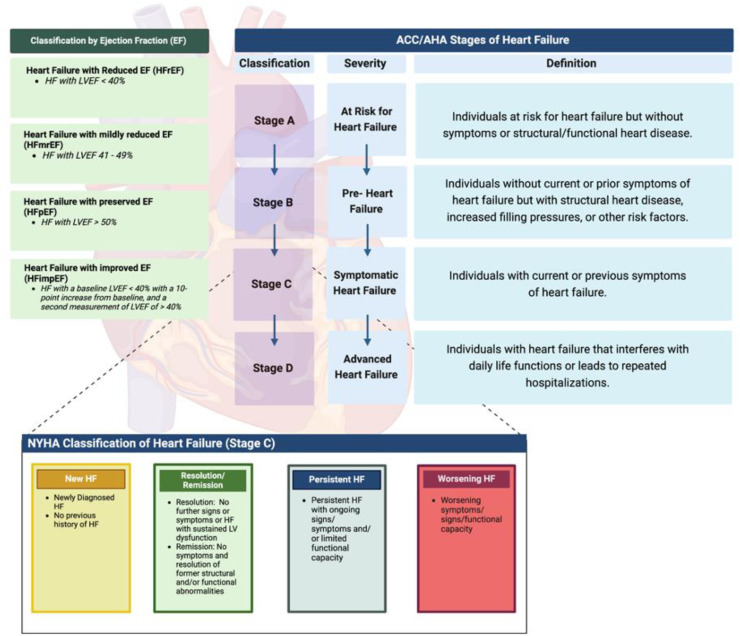
Staging and classification of heart failure. ACC= American College of Cardiology; AHA= American Heart Association; HF=heart failure; NYHA = New York Heart Association; HFrEF = heart failure with reduced ejection fraction; HFpEF = heart failure with preserved ejection fraction; HFmrEF = heart failure with mid-range ejection fraction; HFimpEF = heart failure with improved ejection fraction.

In 2021, HF experts across the globe convened to develop an updated consensus definition of HF to standardize nomenclature in HF research and clinical care [7]. This landmark document emphasized that HF is a clinical syndrome wherein signs and/or symptoms are present in the setting of structural/functional cardiac abnormalities with objective evidence of congestion – represented by either systemic and/or cardiopulmonary findings, or elevation of natriuretic peptide levels. It also revised the nomenclature of the HF staging system, later highlighted in the HF guidelines (Fig. 1). Stage A HF refers to individuals now classified as 'at-risk' for (HF). These patients have predisposing conditions such as hypertension (HTN), diabetes mellitus, coronary artery disease (CAD), obesity, or exposure to cardiotoxic agents, but do not yet have structural heart abnormalities or clinical HF symptoms. Stage B HF, now termed “pre-HF”, describes patients with no symptoms of HF, but evidence of one of the following: (1) structural heart abnormalities (e.g., reduced ventricular compliance, chamber enlargement, ventricular hypertrophy, reduced contractility, or valvular disease); (2) elevated filling pressures detected invasively or non-invasively (e.g., echocardiography); or (3) Stage A risk factors accompanied by elevated B-type natriuretic peptide (BNP) levels or persistently elevated cardiac troponin [5]. Patients with stage C HF, or symptomatic HF, may either experience symptomatic improvement (remission) with medical and device-based therapy or have persistent symptoms despite treatment. Finally, stage D HF represents advanced disease characterized by severe symptoms refractory to guideline-directed therapies. Classification by EF remained as follows: ≤40 defined as HF with reduced EF (HFrEF), 41-49% as HF with mildly reduced EF (HFmrEF) and ≥50% as HF with preserved HF (HFpEF). A new category, HF with improved EF (HFimpEF), was added to include patients with a previously reduced EF that increased by ≥10-percentage points from baseline to more than 40% (HFimpEF). Using this established framework, the present document emphasizes the importance of prevention across a patient’s lifespan, regardless of risk or presence of HF, and irrespective of EF.

## 3 Heart failure epidemiology

HF imposes a major burden on public health, with current prevalence estimates reaching approximately 6.2 million individuals in the U.S., expected to increase to 11.4 million by 2050. HF-related hospitalizations remain high, with nearly 25% of patients experiencing rehospitalization within thirty days of discharge, and 1-year mortality rates approaching 20%[2] HFpEF now comprises nearly 50% of all HF cases. While patients with HFrEF typically face a poorer prognosis, experiencing higher mortality and rehospitalization rates compared to those with HFpEF, outcomes vary depending on the study population and clinical setting [8].

The HFSA annual ‘HF Stats’ document [8] highlights the alarming rise in HF incidence, driven in part by an aging population and the growing burden of comorbidities such as HTN, type 2 diabetes mellitus (T2DM), and obesity. Data from large cohort studies, including Olmsted County [9], Atherosclerosis Risk in Communities (ARIC) [10], Framingham [11], and the Multiethnic Study of Atherosclerosis (MESA) [12], indicate that 56–80% of individuals fall into the “at risk for HF” (Stage A) or “pre-HF” (Stage B) categories. This high prevalence among populations without clinical HF underscores the critical need for proactive prevention strategies, including early risk factor modification and targeted interventions to mitigate HF progression.

## 4 Traditional risk factors for heart failure

**Fig. 2:**
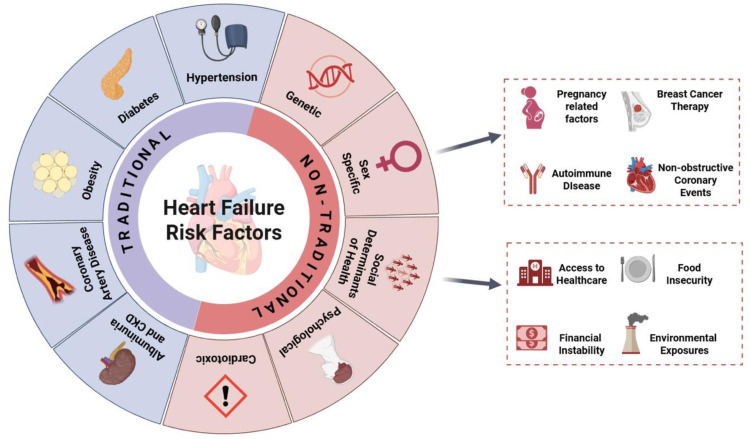
Traditional and non-traditional risk factors for heart failure. Summary of the traditional and nontraditional risk factors detailed in this statement that predispose to the development and progression of heart failure. *CKD = chronic kidney disease*.

Traditional risk factors for the development and perpetration of HF include, but are not limited to, HTN, T2DM, CAD, obesity, and chronic kidney disease (CKD) (Fig. 2). Importantly, beyond potentiating the onset of HF, these risk factors also confer worse prognosis among those with established diagnoses of HF, making their management a crucial component of effective HF management. Select pathophysiologic underpinnings of HF are discussed herein. Guideline recommendations for CVD prevention are shown in Table 1.

**Table 1:** Guidelines recommendations for cardiovascular disease risk reduction.

Risk Factors	ACC/AHA Prevention 2019 Guidelines	ACC/AHA HF Management Guidelines 2022	ADA Guidelines	ESC Prevention Guidelines 2021	ESC HF Guidelines 2021	Consensus
**HYPERTENSION (HTN)** Treatment of HTN is relevant irrespective of stage of HF	All adults with an elevated blood pressure (BP) (120-129/<80 mmHg) or HTN are recommended to follow a heart healthy diet targeting sodium reduction and advised to increase physical activity and limit alcohol consumption. For all adults with Stage 1 HTN (130-139/80-89 mmHg), lifestyle modifications are advised as above. If there is comorbid CKD, DM, or the estimated 10-year CVD risk is >/= 10%, BP lowering medication should be initiated for primary prevention. For all adults with Stage 2 HTN (BP >/=140/90 mmHg) lifestyle modifications and BP lowering medications are recommended.	In patients with HTN at risk for HF, BP should be controlled in accordance with GDMT to prevent symptomatic HF. In patients at risk for HF (including those with HTN), consider BNP/NT-proBNP biomarker screening as part of HF disease prevention. In patients with HFrEF and HTN, up-titration of GDMT, including ARNi/ACEi/ARB, to maximally tolerated dose is recommended.	Individuals with HTN and DM are eligible for anti-HTN pharmacotherapy when BP is greater than 130/80 mmHg with a goal for on treatment BP < 130/80 mmHg. For people with BP > 120/80, lifestyle modifications include the DASH diet, weight loss when indicated, increased physical activity, and moderation of alcohol intake. In patients with DM and HTN who qualify for pharmacotherapy, ACEi or ARB are first line agents.	BP should be classified as optimal (<120/<80 mmHg), normal (120-129/80-84 mmHg), high normal (130-139/85-89 mmHg), Grade 1 HTN (140-159/90-99 mmHg), Grade 2 HTN (160 - 179/100-109 mmHg), Grade 3 HTN (>180/>110 mmHg). For adults with Grade 1 HTN, treatment with pharmacotherapy is based on absolute CVD risk, organ damage, and lifetime benefit. For Grade 2 HTN, pharmacotherapy is recommended. BP goal for all patients is < 140/90 mmHg. For patients between ages 18-69, SBP target range is 120 - 130 mmHg. Preferred medication regimen should include an ACEi/ARB, CCB, or thiazide diuretic.	Treatment of HTN is recommended to prevent or delay the development of HF and limit HF hospitalizations. Medication therapy with ACEi, ARB, beta-blocker, CCB, or thiazide diuretics are recommended	Maintaining a blood pressure below 130/80 mmHg conveys a reduced risk for ASCVD. Interventions for BP control should focus on improving dietary habits with a focus on reduced sodium intake, increasing physical activity and prioritizing weight loss if needed. Pharmacotherapy should be initiated for patients with BP > 140/90 mmHg with an emphasis on the use of ACEi/ARB, thiazide diuretic, or CCB
**OBESITY** Obesity portends an increased risk for HF and CVD. Weight management is essential irrespective of HF stage. For those with ASCVD and/or Stage A HF, the use of incretin therapies should be considered.	In individuals with overweight or obesity, weight loss reduces ASCVD risk. Comprehensive lifestyle counseling surrounding diet, physical activity, and weight monitoring are most effective.	A healthy lifestyle including regular physical activity, maintaining a normal weight, and healthy dietary patterns are recommended.	For patients with obesity and DM, nutrition, physical activity, and behavioral therapy to achieve and maintain >/= 5% weight loss is recommended. Medication assisted weight loss as an adjunct to lifestyle modifications should be considered including the use of diabetic pharmacotherapy that promotes weight loss. Metabolic surgery should be a recommended option for patients with T2DM with BMI >/= 40 and in adults with BMI 35 - 39 who do not achieve weight loss with nonsurgical methods.	Individuals with overweight or obesity should aim for a reduction in weight to reduce BP, dyslipidemia, and decrease risk of T2DM. A healthy diet is the mainstay of weight loss. Bariatric surgery for people with obesity at high risk for CVD should be considered if weight loss cannot be achieved with lifestyle alone.	Counseling against obesity is recommended for primary prevention of HF.	Obesity portends an increased risk for CVD and HF. Weight loss efforts should be comprehensive and focused on improving lifestyle habits, changing dietary patterns, and increasing physical activity.
**DIABETES (DM)** Glycemic control is imperative to prevent the development or progression of HF.	In individuals with T2DM, a heart healthy diet and 150 minutes per week of moderate intensity exercises to achieve weight loss as needed reduces CVD risk. Consideration of metformin as first line pharmacotherapy. Statin therapy should be initiated for people with T2DM who are 40-75 years of age.	In patients at risk for HF (including those with T2DM), consider biomarker screening with a BNP/NT-proBNP in conjunction with GDMT initiation to delay the onset or progression of HF.	In patients with pre-DM, monitor for the progression to T2DM at least annually. Screen for DM based on individual risk assessments. For those with DM, a person centered approach to medication therapy and management is advised. Initiation of combination therapy should be considered.	In patients with T2DM at high ASCVD risk, lipid lowering therapy with LDL-C goal of 70 mg/dL is recommended. Goal LDL-C of 55 mg/dL for those at very high risk or established ASCVD.		Adult patients with T2DM should be on a moderate to high intensity statin regardless of ASCVD risk.
HbA1c	For individuals with HbA1c > 7 despite lifestyle modifications, consider SGLT2i or GLP-1 RA therapies.		An HbA1c goal for adults of < 7 without significant hypoglycemia is recommended. A target HbA1c of < 8 may be considered in patients with limited life expectancy or for those where harms outweigh benefits.	Target an HbA1c goal of < 7.		Targeting an HbA1c goal of < 7 for most individuals to minimize ASCVD risk and progression of microvascular disease is recommended.
SGLT2i In patients with T2DM and ASCVD or at risk for CVD, initiation of SGLT2i reduces the risk of HF progression.	In patients with T2DM and risk factors for CVD, starting an SGLT2i can reduce CVD risk.	In patients with T2DM and CVD or at high risk for CVD, SGLT2i therapy should be initiated to prevent the development of HF. In patients with T2DM with HF or at risk for HF, the use of SGLT2i for glycemic control and reduced HF morbidity and mortality is recommended. In patients with HF, SGLT2i should be utilized regardless of A1C.	For patients with DM, consider switching to an SGLT2i given proven CVD benefits if not already taking. For patients with DM and HF, SGLT2i therapy is advised.	Consider SGLT2i for those with DM at high risk of ASCVD. Start SGLT2i for those with DM and diagnosis of ASCVD.	SGLT2i are recommended for patients with T2DM at high risk of CVD or with CD to prevent progression to symptomatic HF.	In patients with T2DM and ASCVD or at high risk for ASCVD, initiation of SGLT2i reduces the risk of HF progression.
Incretin Therapies For patients with T2DM and CVD or high risk of CVD, initiation of GLP-1 RA reduces the risk of HF progression.	In patients with T2DM and risk factors for CVD, starting a GLP-1 RA can reduce CVD risk.		For patients with T2DM, consider switching to GLP-1 RA given proven CVD benefits if not already taking. For patients with DM and HF, GLP-1 RA is advised.	Consider GLP-1 RA for those with DM at high risk for ASCVD. Start GLP-1 RA for those with DM and diagnosis of ASCVD		For patients with T2DM and CVD or high risk of CVD, initiation of GLP-1 RA reduces the risk of HF progression.
**SMOKING**	All adults should be assessed for tobacco use at healthcare visits. Smoking cessation should be advised to all active tobacco users to reduce ASCVD risk. The use of behavioral therapies and medications for quitting are recommended.	Avoid smoking to reduce future risk of HF. Smoking cessation is advised for individuals with active tobacco use.	Evaluation for tobacco use and referrals for tobacco cessation are recommended for all patients at increased risk of DM.	All tobacco smoking should stop to improve ASCVD risk. Offering of smoking cessation support and pharmacotherapy should be considered.	Smoking cessation is recommended for everyone to reduce the risk of HF.	Adults should be routinely assessed for tobacco use. Smoking cessation is recommended for all patients to reduce risk.
**HYPERLIPIDEMIA (HLD)**	Initiate a moderate intensity statin in adults with an intermediate 10-year ASCVD risk (7.5% to < 20%) for a recommended reduction of LDL-C by 30% or more. For those at high risk, a high intensity statin can be used to lower LDL-C by > 50%. In patients 20 to 75 years of age with LDL-C of 190 mg/dL or higher, maximal tolerated statin therapy is recommended. In adults with DM, moderate intensity statin therapy is indicated regardless of 10-year ASCVD risk.	In patients with a history of MI or ACS, statins should be used to prevent symptomatic HF and reduce ASCVD risk.	Lifestyle modifications focusing on weight loss, incorporating a DASH diet, reducing saturated fat intake, and increasing physical activity are recommended. In adults with DM, it is advised to obtain a lipid profile at the time of diagnosis. For adults with DM, a moderate intensity statin is recommended for primary prevention. A high intensity statin is advised for those with higher ASCVD risks. For patients with DM aged 20-39 with additional ASCVD risk factors, consider initiating statin therapy.	In summary, a high intensity statin up to highest tolerated dose to reach LDL-C goal for a specific risk group is advised. An LDL goal of 55 mg/dL and LDL-C reduction by >/= 50% from baseline should be considered in healthy patients < 70 at very high risk for ASCVD. An LDL goal of 70 mg/dL and LDL-C reduction by >/= 50% from baseline is advised for healthy patients < 70 at high risk for ASCVD In patients with ASCVD, LDL-C goal of 55 mg/dL and a >/= 50% reduction in LDL-C is recommended. For secondary prevention patients not achieving their goal on maximally tolerated statin and ezetimibe, including a PCSK9i is recommended.	Treatment of HLD with statins is recommended for patients at high risk of CV disease or with CV disease to prevent onset of HF.	In patients with elevated ASCVD risk or established CVD, statin therapy is recommended.
**EXERCISE**	Engaging in a minimum of 150 minutes per week of moderate intensity exercise or 75 minutes per week of high intensity physical activity as able.	Daily exercise as part of a healthy lifestyle is recommended.	Physical activity with a goal of 150 minutes per week is recommended for those with DM or those at risk for DM.	All adults should strive for 150-300 minutes of moderate intensity exercise per week or 75-150 minutes of vigorous aerobic exercise per week. For those who cannot meet the above goals, staying as active as their health conditions allow.	Counseling against sedentary habits is recommended for primary prevention of HF.	150 minutes of moderate intensity exercise per week or 75 minutes of high intensity physical activity per week is recommended. For individuals who cannot reach recommended values, prioritizing activity as tolerated is advised.
**DIET**	Emphasizing intake of vegetables, fruits, legumes, nuts, whole grains, and fish to decrease ASCVD risk factors. Reducing intake of saturated fats, foods with high cholesterol and sodium, refined carbohydrates, and processed meats.	A hearty healthy diet is recommended for all individuals at risk of HF.	Medical nutrition therapy is recommended for all patients with DM. Broadly, an emphasis on foods with high micronutrient content including fruits, vegetables, whole grains, lean meats, nuts, and legumes is advised. Minimize consumption of red meat, processed sweets, and refined grains.	Adopting a more plant based and less animal based diet is advised. Limit saturated fats, trans fats, and salt intake. Incorporate daily fiber, fruits, and vegetables. Limit red meat, alcohol, and sugar sweetened beverages.	Following a heart healthy diet is recommended to prevent obesity and reduce risk of HF development.	Following a healthy, predominantly plant based, diet that incorporates lean protein, whole grains, legumes, and nuts. Limiting red meat, processed sweets, saturated fats, and refined grains is recommended.
**CORONARY ARTERY DISEASE (CAD)**	All adults should be routinely screened for traditional cardiovascular risk factors and should have their 10-year ASCVD risk assessed with the pooled cohort equation. For individuals at higher risk, the use of statin therapies is recommended or coronary artery calcium score for those with uncertain risk profiles.	In patients with CVD, optimal management of CVD to prevent progression to Stage B HF. In patients with a history of MI or ACS, beta blockers should be used to reduce mortality.	For patients with T2DM and CVD or at high risk for ASCVD, an SGLT2i and/or GLP-1 RA is recommended. For patients with ASCVD and DM, ACEi or ARB is recommended.	Systemic CVD risk assessment is advised for all individuals with a major vascular risk factor (smoking, family hx of early CVD, PVD, HTN, DM, elevated LDL, obesity, FH).		Routine screening for ASCVD among patients with noted risk factors. In patients with CVD, optimal management of disease is advised, which may include the incorporation of ACEi/ARB, beta blockers, SGLT2i, and GLP-1 RA medications.

### 4.1 Hypertension

It is estimated that a staggering 121,500,000 people live with HTN in the U.S., with important differences in prevalence by race and ethnicity.^1^ HTN is a leading risk factor for the development of HF [1,[13], [14], [15], [16]]. In the Framingham Heart Study (FHS) patient population (n=5143), among patients with newly diagnosed HF, 91% had a pre-existing history of HTN. In this cohort, a diagnosis of HTN was associated with a two to three-fold increase in risk of HF development and, even when controlling for other risk factors, carried the greatest population attributable risk, with a 39% increase in men and 59% increase in women [17].

HTN predisposes to HF through a multifaceted interplay of hemodynamic stress and maladaptive myocardial remodeling. Chronic elevations in blood pressure increase afterload, necessitating compensatory left ventricular hypertrophy (LVH) to maintain cardiac output [18]. Over time, this hypertrophic response leads to myocardial fibrosis, increased stiffness, and impaired relaxation—key drivers of diastolic dysfunction and HFpEF. Additionally, the heightened myocardial oxygen demand due to maladaptive concentric or eccentric remodeling, when combined with microvascular dysfunction and CAD, can induce ischemia and cardiomyocyte apoptosis, thereby promoting systolic impairment and progression to HFrEF [19].

Beyond mechanical strain, hypertension disrupts endothelial homeostasis by reducing nitric oxide bioavailability and promoting oxidative stress and vascular inflammation, which contribute to arterial stiffening [20]. Chronic hypertensive states also trigger neurohormonal activation, notably the renin-angiotensin-aldosterone system (RAAS) and the sympathetic nervous system. These systems foster sodium retention, volume expansion, and further increases in afterload, while also exacerbating myocardial fibrosis and maladaptive β-adrenergic signaling, collectively accelerating the systemic progression of HF [21,22].

Clinical trials have demonstrated that effective blood pressure control significantly reduces HF incidence and adverse cardiovascular events [[23], [24], [25], [26], [27]]. For instance, the Systolic Blood Pressure Intervention Trial (SPRINT) trial reported a 38% reduction in HF incidence and a 23% reduction in all-cause mortality when systolic blood pressure was targeted to <120 mmHg, compared to a target of <140 mmHg [23]. Meta-analyses further support that optimal blood pressure management lowers the incidence of HF diagnoses and events [28,29]. In light of HTN’s pervasive role in HF pathogenesis, adherence to GDMT with a target blood pressure of <130/80 mmHg is critical for HF prevention and equitable cardiovascular care [3].

Selection of pharmacotherapy has also proven to be important, as not all antihypertensive agents have efficacy in reducing the incidence of new HF. Multiple trials have shown the benefit of angiotensin converting enzyme inhibitors (ACEi) in reducing mortality and hospitalizations for patients with clinical HFrEF [[30], [31], [32]]. The Heart Outcomes Prevention Evaluation (HOPE) trial was the first to evaluate the effect of an ACEi on reducing cardiac events in patients at high risk for HF due to the presence of medical comorbidities (Stage A HF). In this randomized controlled trial of ramipril versus placebo, ramipril showed a significant reduction in new HF development (Relative risk [RR] 0.77; 95% Confidence Interval [CI] 0.67-0.87; P<0.001), expanding the benefit of ACEi beyond secondary prevention amongst those with asymptomatic LV dysfunction or symptomatic HFrEF to include primary prevention of HF [33]. The ALLHAT trial looked more broadly to compare effectiveness between classes of antihypertensives, comparing the use of the calcium channel blocker (CCB) amlodipine, the ACEi lisinopril, and the alpha-blocker doxazosin, with the thiazide diuretic chlorthalidone in reducing the incidence of fatal and nonfatal cardiovascular events, with a secondary endpoint of incident HF. While all agents achieved similar rates of blood pressure and cardiovascular event reduction, the doxazosin arm was terminated early due to higher incidence of HF. Compared with patients taking chlorthalidone, patients taking amlodipine had a 38% higher risk of developing HF (Hazard Ratio [HR] = 1.38 [95% CI 1.25–1.52, p<0.001), while those on lisinopril had a 19% higher risk of HF (HR = 1.19; 95% CI 1.07–1.31, p<0.001) [34]. Taken in aggregate, the 2017 ACC/AHA Hypertension Guidelines recommend the use of an ACEi, angiotensin II receptor blocker (ARB), thiazide diuretic, or CCB, either alone or in combination, as primary agents for lowering blood pressure, with the caveat that selection of a specific agent should be based on relevant comorbid conditions [35]. From the perspective of the writers of this joint Statement, selection of antihypertensive therapy should prioritize the prevention of HF, with particular emphasis placed on utilizing ACEi, ARB, or thiazide diuretics as first-line agents.

### 4.2 Diabetes mellitus

An estimated 38.4 million individuals in the U.S.—approximately 12% of the population—are affected by T2DM [36]. T2DM is an important risk factor in the development of HF, and in patients with established HF, the presence of T2DM is associated with increases in cardiovascular mortality and hospitalization rates [37]. Though more prevalent amongst men than women, the presence of T2DM portends a five-fold risk elevation of HF development in women compared to a two-fold increase in men, underscoring significant sex-specific differences in HF susceptibility [38]. Observational data suggest that suboptimal glycemic control further escalates HF risk, and each 5-year increment in diabetes duration is associated with a 17% increased risk of incident HF [39,40].

The effects of T2DM are frequently attributed to its coexistence with comorbid conditions such as HTN, obesity, and ischemic heart disease (IHD), engendering a synergistic constellation of conditions that exacerbate vascular dysfunction, arterial stiffness, and maladaptive cardiac remodeling [41]. In light of these multifactorial risks, comprehensive management of T2DM necessitates an integrated approach that combines lifestyle modifications and stringent blood pressure control alongside optimized glycemic management. While prospective studies have identified hyperglycemia, concomitant obesity, and chronic cardiac strain/volume overload as critical mediators in the progression from subclinical myocardial dysfunction to overt HF [40,42], T2DM also exerts HF risk independent of these factors. In 2013, the ACC and European Association for the Study of Diabetes defined diabetic cardiomyopathy as a pathologic entity characterized by left ventricular dysfunction and impaired myocardial energetics, in the absence of significant HTN and/or CAD [43]. Significant efforts are being made to better understand and address this complex relationship by way of novel agents such as aldose reductase inhibitors aimed at the prevention of clinical HF amongst patients with pre-HF [44].

The study of certain pharmacotherapies intended to achieve improved glycemic control has lent additional insight as to the impact of DM on HF incidence and perpetration. Sodium-glucose cotransporter 2 inhibitors (SGLT2i), for example, were initially proposed for glycemic control but were discovered to confer significant reduction in cardiovascular events, especially HF, amongst patients with T2DM [[45], [46], [47]]. Later, the benefits of these agents to reduce cardiovascular mortality and HF hospitalizations extended to patients with new or established HF in acute or chronic settings, regardless of EF [48,49]. These data have led to the Class 1 recommendation of SGLT2i among patients with T2DM for prevention of HF,[50] as well as the Class 1 (European HF guidelines) and 2a (AHA/ACC HF guidelines) recommendation for reducing cardiovascular death and HF hospitalizations among patients living with HF [51,52]. The recently published Japanese HF guidelines explicitly give both SGLT2i and finerenone a class 1 recommendation for the prevention of HF [53]. (Central Figure)

While the steroidal mineralocorticoid receptor antagonists (MRA) spironolactone and eplerenone have demonstrated mortality benefit for patients with HFrEF [[54], [55], [56]], the nonsteroidal MRA (nsMRA) finerenone was evaluated for the purposes of reducing adverse kidney outcomes in patients with T2DM, demonstrating significant cardiovascular benefits. In the Finerenone in Reducing Cardiovascular Mortality and Morbidity in Diabetic Kidney Disease (FIDELIO-DKD) trial, finerenone resulted in a 14% reduction in the composite of death from cardiovascular causes, nonfatal myocardial infarction, nonfatal stroke, or hospitalization for HF (HR, 0.86; 95% CI, 0.75 to 0.99; P=0.03) among patients with T2DM and severe CKD. This was followed by the Finerenone in Reducing Kidney Failure and Disease Progression in Diabetic Kidney Disease (FIGARO-DKD) trial, which showed similar results in patients with T2DM and less severe CKD, but also demonstrated significant reductions in the incidence of new-onset HF by 32% compared to placebo (1.9% vs. 2.8%; HR 0.68; 95% CI, 0.50–0.93; P=0.0162) [[57], [58], [59], [60]]. These findings underscore finerenone’s important role in mitigating HF risk among patients with T2D and CKD and are the basis for its recommendation in the Kidney Disease: Improving global Outcomes (KDIGO) 2022 Clinical Practice guidelines as well as the American Diabetes Association (ADA) Standards of Care [61].

Incretin-based therapies including glucagon-like peptide 1 receptor agonists (GLP-1RA) have been used to treat T2DM for years, and have demonstrated reduction in the occurrence of CV death, myocardial infarction or stroke [[62], [63], [64]]. On the basis of at least eight CV outcome trials, both cardiovascular and ADA guideline committees recommend treatment with GLP-1RA in patients with T2DM with atherosclerotic cardiovascular disease (ASCVD) or high-risk individuals (patients ≥55years with coronary, carotid, peripheral arterial stenosis >50%, or left ventricular hypertrophy and thereby at risk for HF) regardless of glycated hemoglobin levels (HbA1c) [3]. These agents and their role in preventing HF as well as reducing HF progression are further discussed in the subsequent section.

### 4.3 Obesity

Obesity has reached epidemic proportions globally, and its prevalence is only expected to rise, with a projected increase from 2.6 billion in 2020 to 4.6 billion in 2035 [65]. The U.S. is at particular risk, with an estimated 42.5% of adults over the age of 20 meeting body mass index (BMI) criteria for obesity (as defined by BMI ≥30 kg/m^2^) and 9% meeting BMI criteria for severe obesity (BMI≥40 kg/m^2^) [66]. Importantly, overweight or obesity also manifests as a waist circumference-to-height ratio of over 0.5 and 0.6, respectively, independent of increased BMI, signifying excess or dysfunctional visceral adiposity [67,68]. A recent Lancet Commission has suggested a refinement of how obesity is defined, incorporating how excess adiposity affects the function of organs and tissues [69]. The commission suggests that when obesity is present without evidence of abnormal organ or tissue function, it should be characterized as “preclinical obesity”, while “clinical obesity” is characterized by obesity and the evidence of abnormal organ or tissue function. Thus, the co-presence of CVD and obesity assumes clinical obesity, whereby obesity contributes to the underlying pathophysiological risk of CVD.

Obesity coexists with HTN, hyperglycemia, insulin resistance, obstructive sleep apnea, and CAD - all of which are established risk factors for HF. Previous underappreciation of the direct relationship between obesity and HF may in part have been based on older data demonstrating a “U-shaped” curve relationship between BMI and HFrEF risk and outcomes [70]. In more recent studies, such as a sub-analysis of the Prospective Comparison of ARNI With ACEI to Determine Impact on Global Mortality and Morbidity in Heart Failure (PARADIGM-HF) study, while lower death rates were seen in patients with BMI≥25 kg/m^2^, this relationship was eliminated when adjusting for key variables, including natriuretic peptide levels [71,72]. Studies showing improvement in HF outcomes as a result of weight loss have led to increasing awareness as to the vital role obesity plays not only in increasing the risk of incident HF, but also in portending worse prognosis for those with an existing diagnosis of HF [[73], [74], [75]]. These risks manifest differently in men and women. In a study of more than 20,000 patients across four cohort studies by Savji et al for example, women with obesity were at highest risk for the development of HFpEF whereas men with obesity were at highest risk for the development of HFrEF [76].

**Fig. 3:**
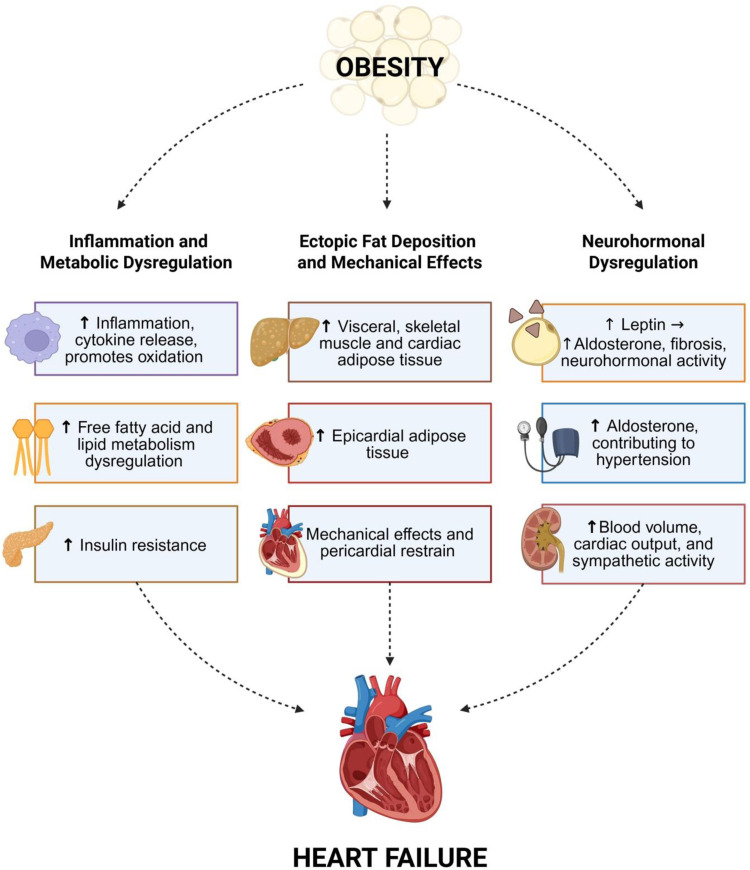
Pathologic underpinnings linking obesity to the development and progression of heart failure. Overview of the multiple pathophysiologic pathways by which obesity contributes to the development of heart failure, including upregulation of inflammatory pathways, increased visceral adipose tissue resulting in cardiac remodeling, and perturbations in the leptin-aldosterone-neprilysin framework.

Obesity independently leads to myocardial dysfunction by way of distinct pathophysiologic mechanisms [68,77] (Fig. 3) Broadly speaking, these fall under the proposed leptin-aldosterone-neprilysin framework [68]. Fat cells secrete adipokines, one of which is leptin – which can promote inflammation, microcirculatory abnormalities, and fibrosis. Aldosterone may be released from adrenal glands in response to leptin or from adipocytes directly. Adiposity is also characterized by increased neprilysin activity, leading to reduced natriuretic peptide levels, exacerbating the interaction of aldosterone and leptin to increase plasma volume, sodium retention, and systemic inflammation and fibrosis [68]. This framework also supports the benefit of MRAs and neprilysin inhibitors in patients with HFpEF, as well as SGLT2i and incretin-based therapies, which reduce excess visceral fat. Specifically, increased metabolic demands lead to increases in cardiac output, wall tension, and dilation of cardiac chambers [78]. Both eccentric and concentric LVH patterns and adverse cardiac remodeling have been observed, contributing to derangements in diastolic function and elevations in intracardiac filling pressures. Myocardial fat deposition coupled with autonomic dysfunction, an increase in circulating catecholamines, and altered heart rate variability lead to inefficient cardiac energetics with elevations in basal heart rates as well [79]. Insulin resistance and altered metabolic profiles with widespread inflammation and hyperglycemia are all seen in conjunction with adipocyte dysfunction, altered lipolysis, and liver steatosis [80]. Mechanical effects of abdominal fat mass on the chest wall lead to increases in intrathoracic pressure, also contributing to increased intracardiac and pulmonary pressures, as well as obstructive sleep apnea and hypoxia [78].

Addressing obesity to mitigate HF is increasingly in focus through a combination of lifestyle and targeted medical therapies. Substantiative data from GLP-1RA cardiovascular outcomes trials have shown a reduced risk of cardiovascular events, including reduced risk of HF, among patients with and without T2DM [81]. The Semaglutide Effects on Cardiovascular Outcomes in People With Overweight or Obesity (SELECT) trial, for example, showed a reduction in HF events with semaglutide compared with placebo among high-risk patients without T2DM [82]. Of the more than 17000 patients with risk factors for or established CVD included, over 13000 did not have a history of HF but were considered “at risk” by virtue of inclusion criteria of age, BMI≥27kg/m^2^ and prior myocardial infarction, stroke, or peripheral arterial disease with claudication and ankle-brachial index <0.85, prior revascularization, or amputation. Patients without HF at enrollment randomized to semaglutide experienced less frequent HF composite endpoint events (HR 0·84, 95% CI [0·74–0·97]). Similar results were seen with tirzepatide among patients with overweight or obesity and without T2DM in the Study of Tirzepatide in Participants with Obesity or Overweight (SURMOUNT-1) [83]. While formal recommendations have not been incorporated into guidelines with respect to use of incretin-based therapies specifically for HF prevention among patients with obesity but without T2DM, accumulating evidence support incorporation in future guideline iterations [[82], [83], [84], [85]].

### 4.4 Chronic kidney disease

Though not often considered a “traditional” risk factor, the authors of this Scientific Statement have deliberately included CKD, particularly in the presence of albuminuria, in this section to emphasize its importance both as a risk factor and potentiator of HF [86]. Albuminuria reflects systemic endothelial dysfunction and intrarenal inflammation, both of which contribute to maladaptive neurohormonal activation, cardiac remodeling, and volume dysregulation—hallmarks of HF pathophysiology. Specifically, the presence of albuminuria >300 mg/d and eGFR <60 mL/min/1.73 m^2^ are associated with incident HF. KDIGO further classifies albuminuria into 3 distinct categories: (normal to mildly increased: <30 mg/g; moderately increased: 30-300 mg/g; and severely increased: >300 mg/g). Data from the Chronic Renal Insufficiency Cohort (CRIC) study showed that elevated urinary albumin-to-creatinine ratio (UACR) was strongly associated with incident HF, even after adjusting for estimated glomerular filtration rate (eGFR) and cardiovascular comorbidities. These findings were corroborated by several other studies, including the RENAAL, FHS, ARIC and MESA, wherein albuminuria consistently conferred an approximately 2-3-fold increased risk of incident HF [11,[87], [88], [89]].

Furthermore, as stated earlier, the FIDELIO-DKD and FIGARO-DKD trials demonstrated a significant benefit (driven in part by a reduction in HF hospitalizations) of finerenone in patients with T2DM and CKD, highlighting the prognostic importance of albuminuria and the therapeutic relevance of targeting mineralocorticoid receptor overactivation [58,90]. These findings reinforce current guidelines that recommend screening for albuminuria as a marker not only of kidney disease progression, but also of heightened cardiovascular risk, including HF, especially in diabetic populations [31,91,92].

Beyond serving as a potent risk factor, the presence of CKD with albuminuria portends a worse prognosis among those patients with established HF. In the Studies of Left Ventricular Dysfunction (SOLVD) trial of patients with HFrEF, the presence of albuminuria conferred a 1.8-fold increased risk of HF hospitalization, an increased risk also observed in the Candesartan in Heart Failure Assessment of Reduction in Morbidity and Mortality (CHARM) trial and GISSI-HF, regardless of T2DM status [86,93,94]. While precise pathophysiological connections between albuminuria and HF are still incompletely understood, common culprit pathways include RAAS activation and systemic inflammation. Observational studies suggest albuminuria may be more commonly encountered in HFpEF; however this remains to be verified. Nonetheless, in the Finerenone in Mildly Reduced or Preserved Heart Failure (FINE-ARTS) study which demonstrated a 16% reduction in the primary outcome of total worsening HF events and CV death over 32 months (rate ratio, 0.84; 95% CI: 0.74 to 0.95; P=0.007) [95], despite a mean UACR of 18mg/g (suggesting normal or mildly reduced kidney function) finerenone still led to a 30% reduction in UACR. This finding suggests that the renal and anti-inflammatory effects of finerenone are applicable across the spectrum of CKD, and that the CKM syndrome framework may benefit from expanded criteria beyond albuminuria alone [96].

Using finerenone as an example, combined data from FIGARO, FIDELIO and FINEARTS emphasize the spectrum of risk (and potential for benefit) that covers a broad range - from those at risk for HF but with significant kidney dysfunction, to those with overt HF but limited evidence of kidney disease as defined by the presence of albuminuria. Efficacy of this nsMRA is currently being investigated among patients with HFrEF in the FINALITY-HF trial for individuals who are intolerant to or not eligible for treatment with a steroidal MRA such as spironolactone or eplerenone. Taken together, though UACR is recommended by KDIGO and ADA among patients with DM to assess risk of CKD progression, its role in predicting HF risk even among patients without DM or CKD is increasingly becoming accepted [86].

### 4.5 Coronary artery disease

IHD is a well-established driver of HF, particularly in cases of HFrEF, where myocardial ischemia and infarction lead to adverse ventricular remodeling and progressive systolic dysfunction. Beyond its role in HF pathogenesis, IHD remains a leading cause of morbidity and mortality, necessitating prevention as an independent clinical priority.

At the critical intersection of prevention and HF management, CAD must be addressed aggressively with a multifaceted approach [97]. First and foremost, targeted lifestyle interventions such as smoking cessation, dietary modification, and structured exercise programs must be emphasized and implemented across the spectrum of risk and disease. Medical therapy for secondary prevention is essential, encompassing optimal blood pressure control, lipid management with high-intensity statins and adjunctive lipid-lowering therapies as indicated, and antiplatelet therapy for those with established ASCVD [97,98]. Additionally, SGLT2i have demonstrated cardiovascular benefits in patients with HF and CAD, further supporting a comprehensive risk-reduction strategy [3].

Early identification of subclinical CAD through coronary calcium scoring, functional imaging, or biomarker assessment may also refine risk stratification and guide individualized interventions [97]. In patients with known CAD and HF, revascularization strategies, either with percutaneous coronary intervention (PCI) or coronary artery bypass grafting (CABG), can be considered based on ischemic burden, myocardial viability, and overall prognosis. Given the complex interplay between CAD and HF, an integrated approach that bridges preventive cardiology, interventional cardiology, and HF care is essential to optimizing patient outcomes [3].

## 5 Non-traditional risk factors for heart failure

As traditional risk factors only account for half of an individual’s lifetime risk of HF, the consideration of nontraditional risk factors aims to create a more comprehensive understanding of HF development. In this section we discuss non-traditional HF risk factors including genetics, sex specific considerations, cardiotoxic exposures (with a focus on cardio-oncology), social determinants of health (SDOH), and psychological and emotional well-being (Fig. 2).

### 5.1 Genetic risk

Rare mendelian genetic variants are well-recognized to be an important contributor to the risk for HF. Genetic testing is frequently performed to establish an etiologic diagnosis, formulate management plans, and stratify prognosis among individuals with suspected inherited cardiomyopathy. However, the increasingly widespread use of whole-exome and whole-genome sequencing has uncovered the existence of such factors in the population at large at an aggregate prevalence of ∼1 in 200 [[99], [100], [101]]. Such variants, with variable prognosis by gene, predispose to the future risk for HF [100,[102], [103], [104], [105]]. The American College of Medical Genetics and Genomics (ACMG) has long recommended the disclosure of incidentally discovered pathogenic cardiomyopathy variants among those going whole-exome or whole-genome testing [106,107]. Reporting may enable early evaluation and surveillance as well as measures toward HF prevention in this high-risk group. Furthermore, the HFSA and ACMG recommend cascade testing to identify at-risk family members to enable early intervention and prevention [108].

The role of common genetic variation is increasingly appreciated in HF risk. Large-scale genome-wide association studies (GWAS) focus on discovering trait-associated genetic variants common in the population. Such variants may be considered in aggregate in the form of polygenic risk scores (PRS) [109]. Given the inherent fixed nature of germline genetic variation, PRS may aid current efforts in the early identification of individuals at elevated risk for HF. GWAS of HF have been limited by imprecise use of HF billing codes, yet have identified several associated genomic loci [110]. Limited assessments of an HF PRS for HF prediction beyond traditional clinical risk factors have not demonstrated significantly improved prediction [111]. More specific multi-trait GWAS of dilated cardiomyopathy (DCM) and left ventricular chamber dimensions have yielded new DCM PRS [112]. The top 10th percentile of the DCM PRS compared to the median had a 3.8-fold risk for DCM [112]. The per standard deviation effect for DCM PRS is 1.6 for DCM and 1.3 for HF [112,113]. Similarly, a HCM PRS is associated with 1.6-fold HCM risk per standard deviation [114]. The clinical utility of these newer cardiomyopathy PRS for HF prediction, in addition to clinical risk factors, is presently not known, meriting further evaluation.

### 5.2 Sex-specific considerations

In addition to traditional risk factors for HF, sex-specific risk factors likely play a significant role in the different risk profiles and manifestations of HF amongst men and women (Fig. 2). Understanding these sex-specific risk factors may help inform individualized risk and prevention strategies. Despite an equivalent lifetime risk of HF between sexes, women have 2.8 times the odds of developing HFpEF, while men have a similar increased odds of development of HFrEF [38]. Importantly, while some traditional risk factors are less prevalent amongst women, when present, the risk of developing HF is substantially higher, such as in the case of T2DM and obesity [38]. An obese phenotype accounts for more than two-thirds of HFpEF broadly, coupled with increased coronary microvascular disease and inflammation, making women at a uniquely higher risk for developing HFpEF compared to men [[115], [116], [117]]. Other sex-predominant factors include a higher prevalence of autoimmune diseases amongst women, higher exposure to anthracycline and tyrosine kinase inhibitor-based therapies for breast cancer, as well as a higher prevalence of non-obstructive coronary events, including stress cardiomyopathy and spontaneous coronary artery dissection [38].

A complete obstetric and gynecologic history is essential for all female patients to understand risk-enhancing factors that contribute to each patient’s unique risk profile of developing HF [38]. Pregnancy offers a unique window to a woman’s cardiovascular risk [118]. Hypertensive disorders of pregnancy, including gestational HTN and preeclampsia, have been associated with increased risk of incident HF [119,120]. In a meta-analysis of 22 studies looking at over 6.4 million pregnant women by Wu et al., the presence of preeclampsia was associated with a 4-fold increased risk of HF (risk ratio, 4.19; 95% CI, 2.09–8.38), an association that continues after adjusting for age, BMI, and T2DM [121]. Gestational HTN in one or more pregnancies is associated with a 1.77 risk ratio of developing HF (1.77; 95% CI, 1.47–2.13) [122]. Other factors that should be taken into account include gestational diabetes, pregnancy loss, premature menopause or ovarian failure, and even age of menarche, which have all been associated with a higher risk of developing CVD and are not included in traditional risk scores [[123], [124], [125], [126]].

Peripartum cardiomyopathy (PPCM) is an idiopathic form of HF characterized by reduced left ventricular EF <45% that presents during the final month of pregnancy or within five months postpartum, in the absence of pre-existing cardiac disease [127]. The etiology is multifactorial, with proposed mechanisms including oxidative stress-induced cleavage of prolactin into a cardiotoxic fragment, angiogenic imbalance, and inflammatory or autoimmune pathways, with emerging evidence suggesting genetic predisposition, particularly *TTN* truncating variants, in select women [128,129]. Established risk factors include advanced maternal age, African ancestry, multiparity, hypertensive disorders of pregnancy (especially preeclampsia), multiple gestation, obesity, and T2DM [127,130]. Given the nonspecific nature of early symptoms—such as dyspnea, fatigue, and edema, intentional screening in high-risk populations is critical. Measurement of natriuretic peptides (BNP or NT-pro-BNP) and echocardiography are essential diagnostic tools to distinguish PPCM from physiologic pregnancy changes [131]. Management includes standard guideline-directed therapy for HFrEF, with adjustments for lactation and reproductive status. Close postpartum surveillance is warranted, as symptoms often arise after hospital discharge. Women with prior PPCM should receive preconception counseling, as recurrent pregnancy carries significant risk of relapse unless there is complete recovery of cardiac function [132]. Risk factor modification, early recognition, and a multidisciplinary, cardio-obstetric approach are central to optimizing outcomes for these patients.

### 5.3 Cardiotoxic exposures

Cardiotoxicity from pharmacotherapy is a common adverse drug effect and can pose a challenge to clinicians when providing patient care. Clinicians should be aware of the potential impact medications can have on the development and trajectory of HF. Mechanistically, medications contribute or exacerbate HF via one or more causes: direct myocardial toxicity, negative inotropic, lusitropic, or chronotropic effects, exacerbating hypertension, delivering a high sodium load, or drug-drug interactions that limit or negate the benefit of other agents that prevent or reduce HF (Table 2A) [133]. In terms of prevention, prioritization of avoiding or limiting drugs with direct myocardial toxicity is prudent (Table 2B) [134]. When selecting medications for treatment, it is critical to evaluate the risks and benefits of the medication alone and in combination with other treatments. This analysis should include consideration of any known risk factors that may predispose or increase risk of myocardial toxicity, as well as any appropriate cardiac testing that should be completed prior to initiation [135]. If risk factors are identified, modifications as to dose, or choice of agents must be tailored accordingly. A team-based approach with a pharmacist can help assure a shared decision for treatment selection, risk factor modification, and medication therapy optimization [136]. Furthermore, it is critical to stay abreast of case reports and/or FDA Medwatch for additional up-to-date information related to cardiotoxicity of various medications.

**Table 2A:** Mechanisms of direct myocardial toxicity^90^.

**Growth Factor Signaling:**Vascular endothelial growth factors, epidermal growth factors, platelet-derived growth factors • Tyrosine kinase inhibitors • Monoclonal antibodies	**Mitochondria functioning:**Fusion-fission cycling and genomic stability • Anti-human immunodeficiency virus therapies • Antibiotics • Chemotherapy agents Apoptosis • Anti-tubulins • Anthracyclines
**Contractility**:Endothelial nitric oxide synthase signaling • Proton pump inhibitors Calcium cycling • Tyrosine kinase inhibitors	**Fibrosis**: • Anti-diabetic agents Central nervous system agents

**Table 2B:** Medications/substances known to cause direct toxicity^91^.

Medications/Substances Known to Cause Direct Mycoardial Toxicity		
5-Fluorouracil	Ergotamine	Mitoxantrone
Amphotericin B	Hydroxychloroquine	Pacitaxel
Anagrelide	Idarubicin	Pergolide
Alcohol	Ifosfamide	Pertuzumab
Bevacizumab	Imatinib	Pioglitazone
Bromocriptine	Interferon	Rosiglitazone
Capecitabine	Interleukin-2	Sorafenib
Chloroquine	Lapatinib	Stimulants
Clozapine	Lenalidomide	Sunitinib
Daunorubin	Lithium	TNF-a inhibitors
Docetaxel	Methysergide	Trasutuzumab
Doxorubin	Mitomycin	
Epirubicin		

### 5.4 Cardio-oncology

It is well established that patients afflicted with cancer are at increased risk of the development of HF by way of increased prevalence of traditional cardiovascular risk factors, shared mechanisms between cancer and CVD [137,138], and increased exposure to potentially cardiotoxic cancer therapies. Prevention of overt cardiomyopathy and HF has focused on: 1) early detection and diagnosis of disease through sensitive diagnostic tools indicative of subclinical injury or stress; 2) control of modifiable risk factors; 3) non-pharmacologic and pharmacologic cardioprotective strategies.

While early changes in echocardiographic measures of cardiac deformation such as global longitudinal strain (GLS) are associated with risk of subsequent declines in left ventricular EF, the Strain Surveillance of Chemotherapy for Improving Cardiovascular Outcomes (SUCCOUR) study demonstrated that use of a GLS-guided strategy of neurohormonal therapy, compared to a traditional left ventricular EF guided strategy, did not result in any significant difference in EF at 3 years post cancer therapy initiation [139]. Similarly, the recently published High-sensitivity Cardiac Troponin I-Guided Combination Angiotensin Receptor Blockade and Beta Blocker Therapy to Prevent Cardiac Toxicity in Cancer Patients Receiving Anthracycline Chemotherapy (CARDIAC CARE) study also suggested a lack of effect of a Troponin-I guided strategy on EF changes at 1 year [140].

Cancer patients also have an increased burden of cardiovascular risk factors that are often under-treated, increasing their risk of incident HF [141]. Thus, there is a general consensus that control and active management of modifiable cardiovascular risk factors in cancer patients is actionable and of clinical importance [137,142]. In addition, considerations towards decreased dose delivery of potentially cardiotoxic therapy (e.g. radiation therapy and decreases in mean heart dose) should be individually tailored [143,144]. With anthracycline-based chemotherapy in particular, dexrazoxane may be implemented for highest risk individuals [145]. Neurohormonal therapy and statins are also used to mitigate cardiovascular risk [146], yet are not recommended ubiquitously given the lack of a substantial, long-term benefit in all-comers or reproducible effects across trials [[146], [147], [148]]. For specific recommendations regarding the prevention and management of cancer-related cardiac dysfunction, refer to the HFSA Statement on Cardio-Oncology [149].

### 5.5 Social determinants of health

SDOH, also known as social drivers of health, including social and community context, neighborhood environment, and access to high-quality health care significantly impact HF risk and outcomes [150,151]. These SDOH, including inability to access high-quality health care, financial instability, environmental exposures, and food insecurity, can contribute to a chronic inflammatory milieu and neurohormonal modulation that enhances HF risk and disease progression [152,153]. Specifically, environmental exposures have strong associations with CVD, but some particularly increase the risk for HF. Air pollution- both ambient and household pollution from biomass fuels- is associated with many forms of CVD, including HF [154]. Arsenic exposure has particularly strong and often irreversible damage to the myocardium [155]. Lead is also cardiotoxic, damaging ion transporters and channels, potentially causing tissue damage and suppressing myocardial contraction [156]. Cadmium exposure has also been demonstrated to be associated with increased levels of galectin-3, a biomarker for myocardial fibrosis, potentially a mechanism for contributing to an increased risk of HF [[156], [157], [158]]. Environmental exposures need to be considered as HF increases, particularly in those living in redlined districts in the US, when environmental risks remain highest [159,160]. This burgeoning body of evidence underscores the need for more comprehensive and practical strategies to address structural and systemic barriers contributing to HF risk and employ interventions to promote equity-focused, risk-based HF prevention.

### 5.6 Psychological and emotional health

Psychological health cannot be considered independent of cardiovascular health, with distinct links noted between depression and anxiety and the incidence of CVD as well as subsequent outcomes (eg, suboptimal self-care, hospital readmissions, and all-cause mortality) [3,[161], [162], [163], [164]]. Though few studies have examined the associations between psychosocial factors and the development of HF specifically, associations with social isolation, loneliness, and work-related stress have been demonstrated [165,166]. A large retrospective study conducted within the MESA cohort (N=6,782), found no association with the presence of psychosocial factors (eg, depressive symptoms, anxiety, anger, hostility and chronic stress) and risk of incident HF [167]. However, these factors may play a role in the incidence of HF in those with a poorer perception of health at baseline, which is particularly relevant for patients residing in historically marginalized and socioeconomically disadvantaged communities [168]. While psychological and emotional health status may not be studied directly in relation to incident HF, there has been ample work connecting psychological conditions such as depression to well-established traditional risk factors for HF, including HTN, T2DM, and obesity, thereby portending increased HF risk. Future study is warranted to better define the role of psychological well-being/health in directly or indirectly conferring HF risk [165,169].

## 6 Risk stratification

**Fig. 4:**
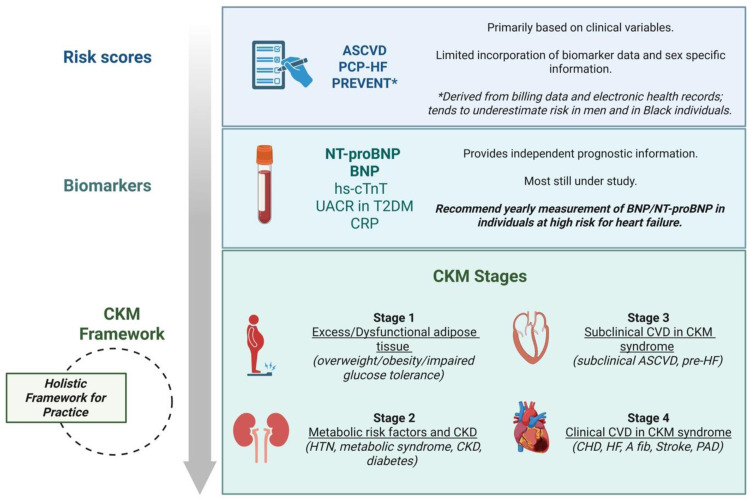
Risk assessment in heart failure. Summary of available risk assessment and stratification tools and an overview of the cardiovascular-kidney-metabolic framework and staging classification. *ASCVD = atherosclerotic cardiovascular disease; PCP-HF = PC equations to Prevent HF; PREVENT = Predicting Risce of Cardiovascular Disease EVENTs score; NT-proBNP = N-terminal Pro-Brain Natriuretic Peptide; BNP = Brain Natriuretic Peptide; hs-cTnT = high sensitivity cardiac troponin T; UACR = urine albumin to creatinine ratio; CRP = c-reactive protein; T2DM = Type 2 Diabetes Mellitus; CKM = cardiovascular-kidney-metabolic; CVD = cardiovascular disease; CHD = coronary heart disease; CKD = chronic kidney disease; A fib = atrial fibrillation; PVD = peripheral vascular disease; HF = heart failure; HTN = hypertension*.

While individual risk factors for the development of HF may be recognized, comorbid conditions often track in tandem, making the quantification of risk challenging, especially when evaluating which patients should receive treatment. Both biomarkers and risk scores provide helpful tools for risk stratification in the clinical setting to determine an optimal therapeutic approach (Fig. 4).

### 6.1 Biomarkers

The understanding and clinical use of biomarkers in HF has evolved dramatically in recent years. The proliferation of fast and reliable laboratory and imaging-based pathologic markers has enabled rapid disease profiling and clinical risk prediction. This is crucial to both primary and secondary prevention in the management of HF. Presently, clinical practice guidelines and consensus documents highlight a role for BNP and NT-proBNP testing as the main driver of biomarker-leveraged early diagnosis of HF risk.

The broad support of testing for BNP or NT-proBNP is in recognition of the fact that when controlling for extensive covariates, the mere presence of a BNP or NT-proBNP above modest thresholds (e.g. BNP >30 ng/L or NT-proBNP >125 ng/L) is associated with heightened risk for progression to symptomatic (Stages C/D) HF and further reduces disparity in HF risk prediction for certain groups (including women and Black individuals). Accordingly, the role of measuring BNP or NT-proBNP for early diagnosis in at-risk populations has robust evidence to support utility for primary prevention strategies, providing opportunity to intervene with lifestyle and therapeutic measures to potentially avert onset of symptomatic stages of HF. For these reasons the American Diabetes Association has joined the ACC/AHA/HFSA in recommending annual measurement of BNP and NT-ProBNP in asymptomatic individuals with T2DM, another high-risk population with substantial rates of unrecognized HF [170].

Although BNP and NT-proBNP now have an established role for prevention of HF onset, it is important to note the limitations and variability in this approach. At the lower concentrations used for early diagnosis of HF risk, factors associated with higher BNP/NT-proBNP (age, female sex, relevant comorbidities such as kidney dysfunction) or lower values (Black race, obesity) must be considered [171,172]. While cut-off points for BNP and NT-proBNP for early diagnosis have utility across larger population analyses, lower values may enhance sensitivity, particularly in categories of patients with lower-than-expected values. Such thresholds are being explored in contemporary studies.

Other biomarkers may have a role for predicting HF risk beyond the natriuretic peptides, including high-sensitivity cardiac troponin (hs-cTn). While originally considered a biomarker of myocardial injury in the setting of acute coronary syndromes, the emergence of refined immunoassays for the measurement of troponin now allows for identification of minute amounts of cardiac injury that in appropriate circumstances may identify heart stress, similar to the information gained from BNP or NT-proBNP measurement. Such abnormalities are linked to risk for incident HF in apparently unaffected individuals in population-based studies. For this reason, concentrations of hs-cTnT or hs-cTnI above the 99th percentile for a normal healthy population are now also considered in the definition of Stage B HF. As with the natriuretic peptides, context matters when interpreting concentrations of hs-cTn for prediction of HF risk; for example, higher values might be expected in men, those with lower socioeconomic status, medical conditions such as T2DM and CKD. Ambiguities remain about whether BNP/NT-proBNP or hs-cTn provide similar or complementary information. Given the broad-based literature supporting BNP/NT-proBNP for early risk prediction, natriuretic peptides are probably preferred; troponin testing may be preferred in situations where BNP or NT-proBNP are ambiguous.

Aligning with current clinical practice guidance, it is the consensus of this committee that a yearly measurement of BNP or NT-proBNP be assessed in individuals at higher risk for incident HF, such as those with LVH, CKD or T2DM. Among those with abnormal values, further evaluation and therapeutic intervention is advised.

Assessment of UACR, indicating albuminuria as discussed earlier, is recommended for individuals with T2DM, as well as those at risk for CKD more broadly, including those with CAD and chronic HTN [173]. Additionally, there are emerging biomarkers for the prediction of risk for incident HF, including those reflecting cardio-renal interactions such as insulin-like growth factor binding protein or apoptosis/fibrosis markers, such as growth differentiation factor-15, the macrophage-based fibrosis biomarker galectin-3, and the interleukin receptor family member, ST2. In each of these cases, these novel biomarkers reflect a cascade spanning from tissue injury, cell-cycle arrest, cellular degeneration, and fibrosis. While these circulating proteins certainly have pathophysiologic relevance, their prognostic and diagnostic capabilities with respect to HF prevention and treatment remain uncertain [174]. Further research will be important in establishing which factors can play a role in guiding preventive strategies.

### 6.2 Role of inflammation

Inflammation is increasingly recognized as a fundamental driver of the pathophysiologic cascade leading to HF, influencing both its onset and progression. Chronic systemic inflammation, characterized by persistent activation of the innate immune response, contributes to endothelial dysfunction, myocardial fibrosis, maladaptive cardiac remodeling, and heightened neurohormonal activation, all of which are implicated in HF pathogenesis [175].

Among inflammatory markers, C-reactive protein (CRP) has emerged as a potential biomarker for HF risk stratification. Elevated high-sensitivity CRP (hsCRP) levels have been associated with increased risk of incident HF, independent of traditional cardiovascular risk factors [176,177]. Prospective cohort studies, including the UCC-SMART (Utrecht Cardiovascular Cohort-Second Manifestations of ARTerial disease) cohort, have demonstrated that in patients with established CVD, CRP is an independent risk marker of incident HF [178]. These data support ongoing trial efforts to assess whether anti-inflammatory agents can reduce the burden of HF. Despite these associations, the integration of CRP into clinical risk models remains limited due to its non-specificity and potential confounding from systemic inflammatory conditions.

The potential of anti-inflammatory strategies in HF prevention and treatment is currently under active investigation. The HERMES trial (NCT05153279), a large-scale randomized controlled study, is evaluating the impact of ziltivekimab, an IL-6 inhibitor, in reducing HF-related events in patients with HFpEF and elevated hsCRP [179]. This aligns with prior evidence from the Canakinumab Anti-inflammatory Thrombosis Outcome Study (CANTOS) trial, which demonstrated that targeted IL-1β inhibition with canakinumab reduced HF hospitalizations, highlighting the role of inflammatory pathways in HF pathophysiology [180]. Other emerging agents, including colchicine and SGLT2i, have demonstrated pleiotropic anti-inflammatory effects that may confer additional cardiovascular benefits beyond glucose and volume control [181,182].

While inflammation is clearly implicated in HF development, its assessment and thereby translation into routine clinical risk stratification remains an area of active exploration. Future studies should focus on defining thresholds for hsCRP elevation that reliably predict HF progression, refining multi-biomarker approaches incorporating inflammatory and cardiomyocyte stress markers, and elucidating which HF phenotypes derive the greatest benefit from anti-inflammatory therapies. As novel targeted anti-inflammatory agents continue to emerge, their role in HF prevention and treatment is poised to expand, potentially ushering in a new era of precision-guided, inflammation-modulating strategies for HF management.

### 6.3 Risk assessment tools

Traditional paradigms to assess CVD risk have been based on the ASCVD risk which do not specifically consider HF. The ASCVD risk score from the Pooled Cohort Equations (PCE) in the U.S. for example, does not consider HF risk in its algorithm [183]. The PCE to Prevent HF (PCP-HF) equation however, specifically assesses HF risk. Derived from 5 racially and geographically diverse US community-based cohort studies of individuals who were free of CVD at baseline, the equation predicts 10-year risk of clinical HF using traditional risk factors in addition to QRS duration [15]. This equation is meant to serve primary care settings but does not incorporate biomarker data and may underestimate risk in certain populations. The new PREVENT^TM^ (Predicting Risk of cardiovascular disease EVENTs) risk score endorsed by the AHA but not yet incorporated into guidelines, has moved beyond ASCVD risk and includes the calculation of risk for HF development, reflecting the recognition of the HF burden and need for early identification and prevention interventions. The score was derived from data of over 6 million patients and 46 datasets and also incorporates markers of impaired kidney function, such as albuminuria and reduced eGFR in its HF algorithm to reflect CKM syndrome stage [184]. Nonetheless, it is important to understand that the PREVENT risk score was derived using billing data and electronic health records from the Optum Data Warehouse [184], and this data remains inaccessible to the public. Critique of the PREVENT risk score has demonstrated that use of this score may underestimate risk, particularly in men and Black adults [[185], [186], [187], [188], [189]].

The recent cardio-kidney metabolic (CKM) syndrome framework endorsed by the AHA categorized in progressive stages (Stages 0-4) reflects the changing landscape of CVD, wherein the systemic nature of CV health and the interconnectedness of organ systems are emphasized [190]. Stage 0 denotes individuals with no risk factors, where Stage 1 CKM represents individuals with excess or dysfunctional adipose tissue and impaired glucose tolerance, Stage 2 begins to point to the web of HTN, hypertriglyceridemia, T2DM, and high-risk CKD. Stage 3 represents subclinical CVD, including ASCVD and pre-HF, and finally, Stage 4 captures overt clinical disease, inclusive of IHD, atrial fibrillation, peripheral arterial disease, stroke, and HF. Using this framework, 90% of the US adult population is estimated to be afflicted with stage 2 or higher CKM syndrome, underscoring the scale of opportunities possible to maximize prevention to mitigate the snowballing progression from risk to disease, and HF specifically.

Risk assessment tools can be useful to identify persons at risk for HF, but need further refinement and validation to understand the impact of embedding prognostic biomarkers like BNP within prediction equations - which to date have not been incorporated into any risk scores. Until this space evolves to capture additional risk, the CKM framework provides for a comprehensive and holistic approach, wherein a more personalized and systemic risk of HF can be assessed.

## 7 Prevention across a patient’s lifespan

Prevention of HF can be considered using the framework of primary, secondary, and tertiary prevention according to the stage of HF. Specific recommendations are shown in the Central Figure, rooted in guideline recommendations, where aquablue signifies higher levels of evidence from randomized controlled trials and yellow-orange represents recommendations and considerations with less robust evidential support, and may be under further validating study. Importantly, these are not meant to supplant societal guidelines, which should be referenced for specific recommendations.

## 8 Lifestyle

The AHA’s Life’s Essential 8 offers a comprehensive framework that underscores the importance of maintaining optimal cardiovascular health through interventions such as maintaining quality sleep, balanced nutrition, regular physical activity, smoking cessation, weight management, and control of blood pressure, cholesterol, and glycemia [191]. This paradigm is not solely reserved for those at imminent risk of developing HF or in the pre-HF stage; rather, its principles are integral across primary, secondary, and tertiary prevention strategies. By universally incorporating these health behaviors into clinical practice, practitioners can not only forestall the development of cardiovascular disease but also attenuate disease progression in patients with established HF, thereby improving long-term outcomes and quality of life. Such an inclusive approach reinforces the notion that the maintenance of cardiovascular health through lifestyle modification is a continuous, lifelong commitment essential for all individuals, regardless of their current disease status [3,191]. Treatment of hypercholesterolemia, blood pressure, obesity, and hyperglycemia remains vitally important as previously discussed. Though ensuring quality sleep and tobacco cessation should be strongly encouraged throughout the patient’s lifespan, here we focus specifically on the role of exercise and rehabilitation, as well as diet in HF. (Central Figure)

### 8.1 Role of exercise and cardiac rehabilitation

Physical activity and exercise are foundational to the prevention of cardiovascular disease and specifically for HF. The United States Preventive Services Task Force recommends that adults 18 years of age and older engage in 150 minutes of moderate-intensity or 75 minutes of vigorous aerobic exercise each week, as well as strengthening exercises at least twice per week [192]. Physical activity has important preventive effects on the development of ASCVD, with direct relevance to HF prevention, given that ischemia remains a primary mediator worldwide in the development of HF [193,194]. While reducing ASCVD is a major benefit of physical activity, there are a myriad additional benefits, including improved parasympathetic regulation, reduction in systemic inflammation, and improved vascular function [195]. Physical activity is associated with a reduction in the development of HF across multiple populations, irrespective of BMI [194,196,197]. and as such the 2022 AHA/ACC/HFSA HF guidelines recommends regular physical activity, in addition to efforts aimed at optimizing nutrition, weight, blood pressure, blood glucose, and avoidance of smoking as keys to preventing the development of HF.^5^

CR is a program constituted by a series of comprehensive and multidisciplinary outpatient interventions to boost physical activity, endurance and strength, with a Class I recommendation for secondary prevention of ASCVD and Class 2b recommendation in patients with stage C HFrEF [3,198]. CR has been shown to lower patients’ risk of ASCVD mortality, reduce one-year hospital readmissions, decrease five-year all-cause mortality, and is cost effective [199,200]. The benefits of CR have been demonstrated repeatedly in multiple populations [201,202]. With supervision, the positive effects of structured physical activity in this setting extend from those at risk to those with pre-existing HF [203]. Mechanistically, there are multiple pleiotropic benefits of CR in patients with HF, including decreased inflammation, improved endothelial function, increased ATP production, and improved peak oxygen consumption [204].

Currently, CR is covered by the Centers for Medicare and Medicaid Services (CMS) for patients with HFrEF (EF <35%) based on data from the Heart Failure: A Controlled Trial Investigating Outcomes of Exercise Training (HF-ACTION) trial [205,206]. In this study of 2331 stable outpatients with HFrEF and NYHA class II-III symptoms, there was a non-significant reduction in all-cause mortality/hospitalization, but significant benefits to health-related quality of life with CR [206]. At the time of writing this document, CR for HFpEF is not covered by CMS or insurances but multiple modestly sized studies have exhibited benefits of CR/exercise training and/or physical therapy interventions in patients with HFpEF in which exercise intolerance is the predominant symptom [207]. For instance, in a pre-specified analysis of the HFpEF subgroup from the Rehabilitation Therapy in Older Acute Heart Failure Patients (REHAB-HF) trial of a novel, multi-disciplinary physical therapy intervention in older patients with acute HF, patients with HFpEF appeared to experience a larger treatment benefit with the intervention in physical performance, exercise capacity, and quality of life [208]. The ongoing REHAB-HFpEF trial is assessing whether this intervention improves clinical outcomes in older patients with HFpEF (NCT05525663).

Despite these proven benefits, CR remains underutilized among many groups, particularly in those with HFrEF [205,209]. Additional studies in varied populations, adjustments to reimbursement, and improved accessibility are crucial to more routine implementation of exercise/rehab routines and programs in the management of patients across the spectrum of risk and overt disease in HF [210,211].

### 8.2 Nutrition

Nutritional interventions constitute a pivotal component in the primary prevention of HF by favorably modulating cardiovascular risk factors such as HTN, obesity, and dyslipidemia. Adherence to dietary patterns—most notably the Dietary Approaches to Stop Hypertension (DASH) and Mediterranean diets—has been shown to reduce blood pressure, enhance endothelial function, and attenuate systemic inflammation, thereby mitigating key pathophysiologic processes underlying HF [212,213]. In general, sodium restriction coupled with increased intake of fruits, vegetables, and whole grains contributes to improved hemodynamic and metabolic profiles, which further decreases the incidence of HF. Among patients with established HF emerging evidence provides insights as to the limits of sodium restriction and challenges conventional paradigms. The Study of Dietary Intervention Under 100 MMOL in Heart Failure (SODIUM-HF) trial, for example, demonstrated that aggressive dietary sodium reduction (<1,500 mg/day) did not significantly improve clinical outcomes compared to usual care and may, in some contexts, provoke adverse neurohormonal responses such as increased renin and aldosterone activation [214]. Conversely, adherence to heart-healthy dietary patterns—notably the DASH and Mediterranean diets—has been associated with improvements in New York Heart Association (NYHA) class, exercise capacity, and reductions in HF hospitalizations, likely due to their favorable effects on endothelial function, oxidative stress, and comorbidity modulation [215,216]. These evidence-based nutritional strategies are endorsed by clinical guidelines for CVD prevention, however recommendations are purposely left broad in the AHA/ACC/HFSA guidelines, with no specific target provided for sodium restriction or endorsement of any one particular diet for the purposes of reducing morbidity and mortality associated with HF [217,218]. What is recommended, however, is the referral to nutrition professionals for tailoring of personalized diet plans, especially for patients with comorbid conditions and/or cachexia. Though the preponderance of data underscores the importance of comprehensive dietary management as a cornerstone in the multifaceted approach to HF prevention and management, further studies are needed to refine which specific interventions are most efficacious for specific populations.

## 9 Primary prevention of heart failure: strategies for patients at-risk and pre-heart failure

Primary prevention strategies are routinely recommended to prevent progression for those at risk for HF (Stage A) or pre-HF (Stage B). (Central Figure) As discussed, these include maintaining healthy lifestyle patterns and managing comorbid conditions [219]. Clinicians can also utilize validated multivariable risk scores to estimate subsequent risk of incident HF (i.e. progression to stage C) in various patient profiles (ACC/AHA class IIa recommendation) [3,[220], [221], [222], [223]]. However, risk prediction tools specifically assessing transition from Stage A to B remain limited in accuracy.

The ACC/AHA/HFSA guideline has given a class IIa recommendation for BNP or NT-proBNP screening in patients at risk for HF [3]. In the ARIC study, incorporating NT-proBNP led to reclassification of 20% of older adults without HF into Stage B [224], highlighting its value in identifying candidates for early preventive interventions.

The majority of the data relevant to prevention of progression beyond pre-HF Stage B stage is in patients with an ischemic phenotype or prior myocardial infarction. For example, SGLT2i are a class I recommendation for patients with T2DM and either established CVD or those at high cardiovascular risk to prevent heart HF hospitalizations [3]. In asymptomatic patients with left ventricular EF ≤40%, ACEi or ARBs, and beta blockers are recommended to prevent symptomatic HF and reduce mortality [3]. Statins are recommended in patients with established ASCVD to prevent symptomatic HF [3]. Coronary revascularization is recommended in some patients with asymptomatic HF in concordance with GDMT. Implantable cardioverter-defibrillators (ICD) are recommended for prevention of sudden cardiac death in patients with left ventricular EF ≤35% who are at least 40 days out from a myocardial infarction and with an estimated >1 year survival and ≤30% among patients with non-ischemic cardiomyopathy [3]. Whether this recommendation will persist in light of modern GDMT may be influenced by the results of the CONTEMP-ICD (Comparative Effectiveness of ICD vs Non-ICD Therapy in Contemporary Heart Failure Patients at a Low Risk for Arrhythmic Death) trial, which will randomize appropriate risk patients with HFrEF to optimal GDMT or GDMT and a primary prevention defibrillator (NCT 06543446) [225]. To this end, clinical trials focused on those patients at-risk for HF but without the clinical syndrome will be vitally important to advance our understanding and practice in HF prevention. The PREVENT-HF study (A Phase III, Randomised, Double-Blind Study to Assess the Efficacy, Safety and Tolerability of Baxdrostat in Combination With Dapagliflozin Compared With Dapagliflozin Alone on Chronic Kidney Disease (CKD) Progression in Participants With CKD and High Blood Pressure) (NCT NCT06268873) is one such international large-scale trial. Baxdrostat (an aldosterone synthase inhibitor) in combination with dapagliflozin will be studied in a randomized fashion amongst patients at risk for HF.

## 10 Secondary prevention after heart failure diagnosis

Effective post-HF management necessitates a comprehensive, multidisciplinary approach as the vast majority of patients with HF present with a constellation of comorbid conditions, such as HTN, T2DM, atrial fibrillation, and obstructive sleep apnea, that, when inadequately controlled, contribute to increased morbidity and mortality. These conditions are not merely coexisting pathologies but integral components of the HF syndrome, necessitating a coordinated treatment strategy that prioritizes evidence-based pharmacologic and non-pharmacologic interventions.

At the core of secondary prevention is the optimization and up-titration of GDMT, which has been shown to significantly alter the disease trajectory in HF. In HFrEF, current guidelines strongly recommend quadruple therapy, including a RAAS inhibitor (angiotensin receptor–neprilysin inhibitor [ARNI], ACEi, or ARB), a beta-blocker, an MRA, and an SGLT2i. As previously mentioned, for HFpEF, SGLT2i have emerged as the first class of medications with a Class 1 guideline recommendation in the European guidelines but a 2a in the AHA/ACC/HFSA guidelines [48,226].

In the near future, emerging therapies such as nsMRA and GLP-1RA may be integrated and endorsed more broadly in HF care. Compared with steroidal MRAs, finerenone offers increased receptor selectivity, a potentially lower risk of hyperkalemia, and exhibits anti-inflammatory and antifibrotic properties, supporting its role as a key component of future preventative and therapeutic strategies. Planned trials will determine its utility in HFrEF. In parallel, growing evidence supports the utility of incretin-based therapies in HF, particularly HFpEF. GLP-1RAs, as described above (*see Obesity section*), have shown significant cardiometabolic benefits, not only through glycemic control and weight reduction, but also by attenuating the progression of HF independent of T2DM or weight loss. Although current guidelines do not formally endorse incretin-based therapies for patients without diabetes, given the accumulating evidence, future recommendations are likely.

In addition to pharmacologic therapy, a heart‐healthy, low-sodium diet has been considered a cornerstone of HF management, as it is generally thought to alleviate volume overload, improve hemodynamics, and enhance the efficacy of pharmacologic therapies. Clinical studies, however, have been inconclusive in the demonstration of sodium restriction leading to fewer hospitalizations or improved survival [214]. In parallel, CR has been shown to enhance functional capacity, reduce rehospitalization rates, and lower overall mortality [203,[227], [228], [229], [230]]. Further work is needed to demonstrate improved outcomes for patients across the EF spectrum to influence broader coverage and access to this effective therapy. (Central Figure)

## 11 Tertiary prevention

While primary and secondary prevention recommendations have been the subject of extensive study, how to manage patients after heart replacement therapy is less well established. Herein, the term tertiary prevention is used to capture the essential management of risk factors (more often referred to as comorbid conditions at this stage) and the preventive nature of care delivered for this population. Tertiary prevention, therefore, is defined here by the preventive therapies that warrant consideration among those patients living with a left ventricular assist device (LVAD) or heart transplant.

### 11.1 Post left ventricular assist device (LVAD) considerations

The preventive interventions following LVAD implantation are critical to optimizing long-term outcomes and preserving optimal eligibility for heart transplantation. Given that many patients with LVADs harbor multiple comorbid conditions—such as HTN, T2DM, and obesity—that exacerbate cardiovascular risk and adversely impact overall prognosis, a holistic management strategy is imperative [231,232]. Rigorous blood pressure control minimizes vascular shear stress and prevents adverse remodeling, while meticulous management of glycemic status and weight—often utilizing novel incretin-based therapies—mitigates systemic inflammation and improves metabolic efficiency [[232], [233], [234]]. Recent studies in this population illustrated improvements in cardiometabolic profiles for patients on GLP1-RAs, with significant reductions in weight, NT-proBNP, and HbA1C levels observed [235]. This integrated approach not only reduces the incidence of device-related complications and hospital readmissions but also enhances end-organ function, thereby increasing the likelihood of successful transplantation in a well-selected patient population. (Central Figure)

### 11.2 Post heart transplant management considerations

Tertiary prevention following heart transplantation is paramount to mitigate the risk of coronary allograft vasculopathy (CAV) and other cardiovascular complications that can compromise long-term outcomes. The International Society for Heart and Lung Transplantation (ISHLT) guidelines advocate for rigorous cardiovascular risk management, specifically recommending that blood pressure be meticulously controlled and low-density lipoprotein (LDL) cholesterol maintained at levels below 100 mg/dL through the routine use of statin therapy [236]. Novel applications for proprotein convertase subtilisin/kexin type 9 (PCSK9i) in this patient demographic have also yielded promising results, demonstrating significant reduction in LDL levels in patients with uncontrolled hyperlipidemia and reduced incidence of CAV. These benefits may derive from the immunomodulatory effects of PCSK9i, specifically via the prevention of proinflammatory cell chemotaxis and downregulation of inflammatory signaling pathways [237]. In addition, vigilant efforts to prevent and manage post-transplant T2DM—through regular metabolic monitoring and the promotion of healthy lifestyle behaviors, including dietary modifications, weight management, and structured physical activity—are critical. Areas of ongoing research include the use of SGLT2i and GLP-1RAs in heart transplant recipients, with small retrospective studies yielding positive results with observed reductions in weight, blood pressure, and HbA1c [238,239], in addition to lower rates of transplant rejection in those with T2DM [240]. Still, their widespread use remains limited, potentially due to the perceived risk of genitourinary infection development in this immunosuppressed population. Currently, multiple prospective trials are underway exploring the efficacy and safety of SGLT2i in improving glycemic control and their impact on renal function in transplant recipients [237]. Overall, an integrated, multidisciplinary approach to combat CKM disease not only attenuates the progression of CAV but also enhances overall graft survival and patient longevity, underscoring the imperative for comprehensive tertiary prevention in this vulnerable population [236]. (Central Figure)

## 12 Multidisciplinary collaboration

**Fig. 5:**
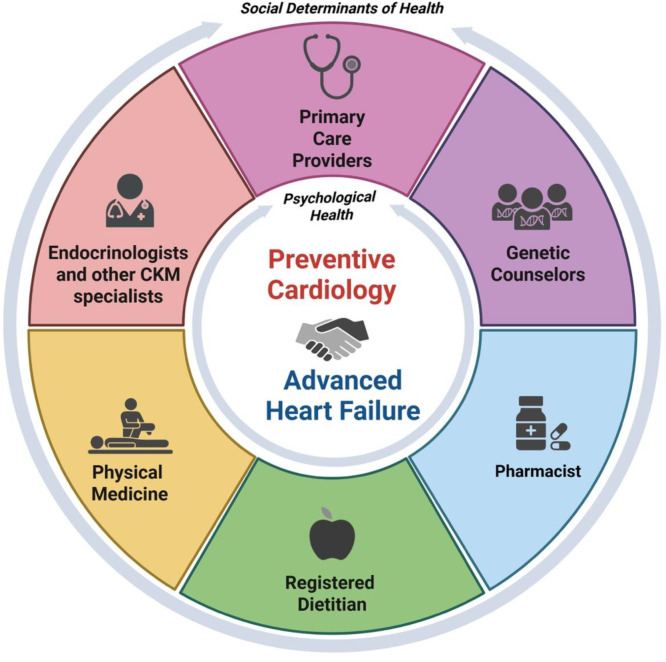
Multidisciplinary partnerships and holistic care required in the prevention of heart failure.

HF frequently serves as the end road for CKM [241]. With expanded options for HF treatment and a strong emphasis on prevention, a collaborative, integrative model that bridges together various specialties (within and outside of cardiology) to optimize patient care and improve outcomes is needed. This joint Scientific Statement committee advocates for specialized HF Prevention clinics that have the ability to bring together various healthcare providers to facilitate seamless communication and comprehensive patient care (Fig. 5). These clinics can also function as centers of education and research, advancing best practices. Collaboration between preventive and HF specialists is essential for identifying patients at risk of developing clinical HF and preventing disease progression by the timely application of appropriate medical therapy [3]. By working together, these specialists can design comprehensive screening protocols that employ advanced diagnostic tools and risk assessment models. The cardiovascular preventive clinician leverages their expertise in managing cardiovascular risk factors and promoting lifestyle modifications, while the HF specialist plays a crucial role in optimizing treatment strategies for patients in more advanced stages of the disease. Other cardiovascular specialists are brought in as appropriate, such as electrophysiologists managing tachyarrhythmias and interventional cardiologists treating acute coronary syndromes. A collaborative approach between cardiovascular subspecialities not only enhances early intervention efforts but also ensures a continuum of care that addresses both prevention and management across the spectrum of HF.

The rising prevalence of HF among the elderly, as well as interrelated cardiometabolic conditions that share pathophysiology and treatment avenues requires the involvement of various specialists beyond cardiology alone [242]. Endocrinologists, for example, are instrumental in managing conditions like T2DM and obesity, and may be more comfortable with more nuanced management [3]. With expert input from specialists, dieticians, pharmacists, and exercise physiologists, among others, cardiometabolic clinics could serve as HF Prevention hubs, facilitating comprehensive coordination of HF preventive efforts. Assessment of specific genetic mutations known to predispose to HF is another important consideration, where early detection can lead to preemptive measures [108]. This proactive approach may go beyond aggressive management of traditional and non-traditional risk factors to include early genetic testing and phenotyping, frequent monitoring, early initiation of targeted therapies, and screening of family members to mitigate the identified risks. Moreover, integrating genetic and cardiometabolic profiling could enhance risk identification and allow for personalized preventive care.

Given the need to focus proactively on early HF detection, increased awareness and collaboration with primary care clinicians is paramount [3]. By providing these clinicians with the necessary expertise and tools, such as validated risk assessment instruments, the timely recognition and intervention for HF could be improved, thereby enhancing prevention and facilitating coordinated care referrals. The opportunity to start preventive strategies is also present in the inpatient setting, especially for those patients who do not seek outpatient care regularly. The identification and initiation of preventive strategies for hospitalized patients being treated for other cardiac and non-cardiac conditions offers an important window for the implementation of strategies early on in the natural history of HF. This approach could expedite referrals to specialized care months or even years before symptoms begin to manifest.

## 13 Digital health

### 13.1 Integration of artificial intelligence in HF prevention and management

Advancements in health technology are reshaping HF prevention by enabling a personalized, continuous care model that replaces traditional episodic care. Central to this transformation is the integration of artificial intelligence (AI), which leverages predictive analytics to anticipate clinical deterioration and guide proactive intervention. AI and its subset, machine learning, emulate human decision-making by iteratively learning from complex data patterns to refine diagnostic and therapeutic strategies [243,244]. The implementation of AI carries the potential to improve the prevention and prediction of HF on multiple fronts, such as early and enhanced diagnosis, improved risk prediction, and remote monitoring and management. AI-based models for HF diagnosis, for example, include using the combination of ECG, echocardiogram, and electronic health data [[245], [246], [247], [248]]. HF is a clinically heterogeneous syndrome, and AI can help identify novel subgroups of patients with distinct phenotypes, which may have differing treatment responses or risks of disease progression [[249], [250], [251]]. AI-leveraged healthcare technology could improve the precision of HF detection and personalization of targeted therapies.

A key question is the ability of AI to outperform traditional cardiovascular risk prediction tools. The 2023 AHA PREVENT risk score for total CVD, HF, and ASCVD chose not to use an AI approach because the score focused on established risk factors with well-understood risk gradients and age-specific interactions [184]. AI may therefore add value when numerous risk factors with unknown or nonlinear interactions are included. An underexplored area in personalized risk prediction is for algorithms that learn an individual’s baseline over time and can make assessments based on what is normal or abnormal for an individual, rather than ubiquitous diagnostic cut-offs. It must be ensured that models are trained and validated across a diverse dataset to prevent biases from being integrated population-wide, that data sharing is standardized to create the strongest models, and that health systems promote opportunities for rethinking traditional care models to integrate novel AI pathways [252].

### 13.2 Wearable devices/health and fitness trackers

Devices and sensors (e.g. smartwatches, patch monitors, apps) engage individuals in their own health and enable clinicians to evaluate trends and identify actionable insights using metrics like heart rate and rhythm, respiratory rate, physical activity, body posture and position, blood pressure, weight, sleep, blood glucose, and more [253]. While some sensors may have applicability for screening in the general population, wearables specific for HF may improve outcomes for select patients. For example, in the LINK-HF (Multisensor Non-invasive Remote Monitoring for Prediction of Heart Failure Exacerbation) trial, a remote telemetry multisensor chest patch paired with a smartphone-based AI algorithm detected increased risk of hospitalization in HF patients with 76-88% sensitivity, 85% specificity, and a median time between alert to readmission of 6.5 days [254]. Management based on implantable wireless pulmonary artery pressure monitoring has also been shown to reduce HF hospitalizations [255]. Challenges to scaling wearables in practice include device accuracy, cost, patient compliance, and deriving actionable data from large data lakes. Advances in electronics have led to smaller wireless sensors which promote wearable use and implantable monitoring.

CR is well established as a key program for secondary prevention of cardiovascular disease by delivering education, medication adherence promotion, risk factor management, nutritional counseling, psychosocial support, and structured exercise (See section above on CR). Although in-person completion of CR has been shown to lower mortality, secondary events, readmissions, and hospitalizations as well as improve functional status and quality of life for patients with HF, many barriers to center-based CR participation exist resulting in overall low rates of engagement [256,257]. These traditional center-based models may be limited by SDOH for example, including transportation, program availability, and cost, which create barriers to ubiquitous access for underrepresented communities [258]. Home-based virtual CR programs have shown promise in overcoming known access barriers to center-based CR by safely delivering components of CR virtually. Multiple studies have demonstrated the feasibility and potential for success of digital home-based CR and ongoing trials supported by the AHA continue to evaluate promising virtual CR models (mTECH trial) [259].

It is anticipated that digital health and leveraging AI will be key in optimizing HF prevention, diagnosis, and disease management in the future. These technologies will acquire more complex abilities and serve as a core component of the HF prevention armamentarium in clinical practice.

## 14 Spirituality, wellness, and holistic health

Within cardiology and the medical field in general, areas of wellness such as spirituality, resilience, well-being, meditation, and holistic health remain under-discussed and under-utilized. This is despite the AHA highlighting psychological health and well-being as foundational factors of cardiovascular health, as well as consistent data on the cardiovascular benefits of these activities [165,191,260]. For patients with established HF, a population with frequent concomitant depression and psychosocial stress, this is particularly important [261]. Providing clear and communicable avenues by which patients and providers can access the benefits of these therapies, therefore, becomes of great importance.

Exposure to stress, traumatic situations, and negative psychological health is clearly associated with worse cardiovascular outcomes and CVD risk [165,262]. In the HF population, depression is common and independently associated with worse cardiovascular outcomes [263]. Screening for anxiety and depression using brief in-office questionnaires allows providers to identify these conditions and improve outcomes through healthy practices and referrals to allied health professionals [264].

Meditation is one mindfulness practice readily available to all patients. Prior studies on the benefits of meditation in the management and prevention of cardiovascular disease have produced modest results, suggesting possible benefit in risk reduction. In meta-analysis and systematic review of meditation and HF, a small number of studies have shown improved quality of life metrics and reduced HF symptoms [265,266]. Clearly defining and modeling meditation interventions for patients should be an important area of focus for practitioners, with future studies examining best practices in robust randomized clinical settings, especially in the prevention of heart disease and HF specifically.

Religiosity and spirituality have further been demonstrated to improve markers of cardiovascular wellness [267]. Use of tools like the Faith Importance Influence Community and Address (FICA) spiritual history tool to assist clinicians, patients, and their families to share their spirituality and allow them to better cope with their illness should be evaluated in those living with HF [268]. For patients in the final stages of HF, palliative care consultation is recommended across society guidelines, with spirituality being a core component of palliative care practice [3,269]. Despite this, spirituality remains understudied in HF as compared to other clinical domains, such as oncology [268]. Additional work defining and incorporating spirituality in the care of HF patients remains a vital need within this field.

## 15 Language matters: moving from failure to function

Language in HF is not merely a medium of communication but a potent determinant of patient engagement, self-perception, and therapeutic outcomes. The terminology clinicians use—from “heart failure” to descriptors of disease severity—can either empower patients or inadvertently engender feelings of defeat and stigma [[270], [271], [272]]. By adopting patient-centered language that emphasizes heart function and resilience rather than inevitable decline, clinicians can foster improved adherence, shared decision-making, and overall satisfaction with care [273]. Moreover, precise and empathetic communication is essential for aligning clinical goals with patient values, thereby enhancing both the scientific rigor and the humanistic dimensions of HF management.

## 16 Conclusions

Realizing a transformative vision in HF care—one that begins with prevention at its earliest stages—requires healthcare systems and clinicians to lead with intention, embracing innovation and fostering interdisciplinary collaboration. Central to this paradigm shift is the prioritization of preventive strategies, which will necessitate systemic changes in policy, clinical practice, and care delivery models. Clinicians must be equipped and empowered to adopt a prevention-focused mindset, integrating current guidelines and validated risk models into routine practice. This includes early initiation of evidence-based therapies in asymptomatic, high-risk individuals and thoughtful evaluation of emerging interventions. Researchers are uniquely positioned to accelerate progress through advances in genomics, AI, and big data, enabling more precise risk stratification and discovery of novel therapeutic targets. Continued investment in elucidating the genetic, inflammatory, and metabolic drivers of HF will be critical to evolving prevention frameworks. For policymakers and health system leaders, removing barriers to the timely implementation of GDMT across the spectrum of risk is imperative. This includes expanding access to specialized HF and cardiometabolic clinics and integrating technology-enabled, personalized care, particularly for underserved populations.

The future of HF care lies in proactive, not reactive, intervention. Through coordinated, multidisciplinary efforts that leverage predictive analytics, advanced therapeutics, and equitable care models, the medical community can fundamentally alter the trajectory of HF and redefine the standard of cardiovascular care.

## Funding

None.

## CRediT authorship contribution statement

**Anuradha Lala:** Writing – original draft, Writing – review & editing, Conceptualization. **Craig Beavers:** Writing – review & editing, Conceptualization. **Vanessa Blumer:** Writing – review & editing, Conceptualization. **LaPrincess Brewer:** Writing – review & editing, Conceptualization. **Diana De Oliveira-Gomes:** Writing – review & editing, Conceptualization. **Sandra Dunbar:** Writing – review & editing, Conceptualization. **Hannah Every:** Writing – review & editing, Conceptualization. **Richard Ferraro:** Writing – review & editing, Conceptualization. **Bonnie Ky:** Writing – review & editing, Conceptualization. **James Januzzi:** Writing – review & editing, Conceptualization. **Francoise Marvel:** Writing – review & editing, Conceptualization. **Robert Mentz:** Writing – review & editing, Conceptualization. **Erin Michos:** Writing – review & editing, Conceptualization. **Jagat Narula:** Writing – review & editing, Conceptualization. **Khuram Nasir:** Writing – review & editing, Conceptualization. **Pradeep Natarajan:** Writing – review & editing, Conceptualization. **Lori Ann Peterson:** Writing – review & editing, Conceptualization. **Fatima Rodriguez:** Writing – review & editing, Conceptualization. **Michael D. Shapiro:** Writing – review & editing, Conceptualization. **Jenna Skowronski:** Writing – review & editing, Conceptualization. **Randall C. Starling:** Writing – review & editing, Conceptualization. **Pam Taub:** Writing – review & editing, Conceptualization. **Ryan J. Tedford:** Writing – review & editing, Conceptualization. **Quentin Youmans:** Writing – review & editing, Conceptualization. **Shelley Zieroth:** Writing – review & editing, Conceptualization. **Martha Gulati:** Writing – original draft, Writing – review & editing, Conceptualization.

## Declaration of competing interest

P.N. reports research grants from Allelica, Amgen, Apple, Boston Scientific, Genentech / Roche, and Novartis, personal fees from Allelica, Apple, AstraZeneca, Bain Capital, Blackstone Life Sciences, Bristol Myers Squibb, Creative Education Concepts, CRISPR Therapeutics, Eli Lilly & Co, Esperion Therapeutics, Foresite Capital, Foresite Labs, Genentech / Roche, GV, HeartFlow, Magnet Biomedicine, Merck, Novartis, Novo Nordisk, TenSixteen Bio, and Tourmaline Bio, equity in Bolt, Candela, Mercury, MyOme, Parameter Health, Preciseli, and TenSixteen Bio, and spousal employment at Vertex Pharmaceuticals, all unrelated to the present work.

M.G. is supported by contracts from the National Heart, Lung, and Blood Institutes nos. N01-HV-068161, N01-HV-068162, N01-HV-068163, N01-HV-068164, grants U01 HL064829, U01 HL649141, U01 HL649241, K23 HL105787,K23 HL125941, K23 HL127262, K23HL151867, T32 HL069751, R01 HL090957, R03 AG032631, R01 HL146158, R01 HL146158-04S1, R01 HL124649, R01 HL153500, U54 AG065141, General Clinical Research Center grant MO1-RR00425 from the National Center for Research Resources, the National Center for Advancing Translational Sciences Grant UL1TR000124, Department of Defense grant PR161603 (CDMRP-DoD), and grants from the Gustavus and Louis Pfeiffer Research Foundation, Danville, NJ, The Women’s Guild of Cedars-Sinai Medical Center, Los Angeles, CA, The Ladies Hospital Aid Society of Western Pennsylvania, Pittsburgh, PA, and QMED, Inc., Laurence Harbor, NJ, the Edythe L. Broad and the Constance Austin Women’s Heart Research Fellowships, Cedars-Sinai Medical Center, Los Angeles, CA, the Barbra Streisand Women’s Cardiovascular Research and Education Program, Cedars-Sinai Medical Center, Los Angeles, CA, The Society for Women’s Health Research, Washington, D.C., the Linda Joy Pollin Women’s Heart Health Program, the Erika Glazer Women’s Heart Health Project, the Adelson Family Foundation, Cedars-Sinai Medical Center, Los Angeles, CA, Robert NA. Winn Diversity in Clinical Trials Career Development Award (Winn CDA), and the Anita Dann Friedman Endowment in Women’s Cardiovascular Medicine & Research. This work is solely the responsibility of the authors and does not necessarily represent the official views of the National Heart, Lung, and Blood Institute, the National Institutes of Health, or the U.S. Department of Health and Human Services. *Consultant Fees/Honoraria:* Novartis, New Amsterdam and Medtronic Inc, unrelated to this work.
